# Supplementary material for: The 2016 California policy to eliminate nonmedical vaccine exemptions and changes in vaccine coverage: An empirical policy analysis
Source: PLoS Med. 2019 Dec 23;16(12):e1002994. doi: 10.1371/journal.pmed.1002994 (PMC6927583; doi:10.1371/journal.pmed.1002994)
Supplement: S1 Appendix — (DOCX) [file pmed.1002994.s001.docx]

**S1 Appendix: The Synthetic Control Method and State Level Data**

*State level data*

State level coverage and exemption data were retrieved from the CDC’s SchoolVaxView website [1]. Data was available for the 2009 - 2010 school year to the 2017 - 2018 school year. Data for the 2010 - 2011 school year was unavailable because it was not verified and so only data from 2011 onwards was used in this analysis. State level characteristic data was retrieved from various sources. All data and their sources are listed in S1 Table.

*Synthetic control methodology*

The synthetic control approach creates a synthetic control California that describes the change in the outcome in the absence of the treatment. The synthetic California is constructed from a weighted combination of all potential control states. The synthetic control optimization algorithm chooses a set of weights that minimize the difference between the actual California and the “synthetic” California in the pre-treatment period. The resulting synthetic control California provides a counterfactual estimation of the study outcome during the post-policy period in the treated state in the absence of the treatment.

We defined three outcomes for the state level analysis: MMR coverage, prevalence of non-medical exemptions, and prevalence of medical exemptions. In order to be included in the pool of potential control states for a given outcome, a state needed to have complete data records for all study years for that outcome. If a state was missing any data for any year for an outcome it was excluded from the control pool for that outcome. The states excluded from the control pools for each outcome are listed in S2 Table. Since the synthetic control method minimizes the difference in the outcome between the synthetic California and the actual California in the pre-treatment period, we created three synthetic “California” states, one for each outcome as per common practice.

*Covariate Selection and Cross Validation*

We identified an initial set of potential characteristic covariates via a review of the literature and expert consultation. We then used variable selection and cross-validation to select the final set of covariates to include in the model for each outcome. We selected an initial set of 15 demographic and health related state characteristic covariates. Covariates and data sources are shown in S1 Table.

We conducted the variable selection and cross validation as follows. We split the pre-policy vaccine coverage data (2011 - 2015) into a training set (2011, 2012 and 2013) and a testing set (2014 and 2015). We then created a synthetic control California using only the training subset of the data. We compared the synthetic control California to the treated California testing subset of the data (2014 and 2015). We defined a Root Mean Square Predictive Error (RMSPE) to measure how well the synthetic control California matched the treated California for the 2014 - 2015 testing period (in the absence of a policy change, the synthetic control California should match the treated California, i.e. RMSPE should be minimized).

We repeated this process with different covariate combinations, using a stepwise variable addition procedure. We chose the variable combination that minimized the test RMSPE. The RMSPE values for different covariate combinations, as well as the cut offs for each outcome, are shown in S1 Figure.

For the state level MMR outcome, we created the synthetic control by matching on the following final set of characteristic covariates:

i) Median Age (yrs),

ii) Children with no well child visits (%),

iii) Uninsured (%),

iv) Private insurance (%).

For the state level medical exemptions outcome, we created the synthetic control by matching on the following final set of characteristic covariates:

i) Private insurance (%),

ii) Per capita health spending ($).

For the state level non-medical exemptions outcome, we created the synthetic control by matching on the following final set of characteristic covariates:

i) Median Age (yrs),

ii) Population (people),

iii) Education below high school level (%),

iv) No coverage (%).

For each outcome we also included the average value of all pre-policy outcome values as a lag covariate, following the convention set by Abadie et al. in their analysis [2-4]. The resulting weights of each of these covariates are shown in S3 Table.

*Placebo Testing*

We conducted a set of placebo tests (also known as permutation tests) to evaluate whether the changes in coverage observed for California were meaningful relative to the changes in coverage in the untreated states. Placebo tests are commonly used in synthetic control analyses for inference and are akin to statistical hypothesis testing. To conduct the placebo tests, we iteratively reassigned treatment status to every state in the control pool. We then created a synthetic control for each of these states using all other states (including California) as control states. For each state, we then re-calculated an effect size. The effect size was calculated by finding the difference in coverage between the first two years post policy (2016 and 2017) and the last two years pre-policy (2014 and 2015) for both the treated state and the synthetic control. The difference for the synthetic control was then subtracted from the difference for the treated state. This effect size was compared between all states. Since California was the only treated state (i.e. it was the only state with a new policy), we expected it to be the state with one of the largest effect sizes. To account for baseline variability in state vaccination coverage, we pre-specified a meaningful relationship as having an effect size in the top 5th percentile of effect sizes. This process was repeated for all three outcomes and results are shown in Figure 3 of the main analysis.

*Sensitivity analyses*

We performed a set of sensitivity analyses to determine the robustness of our findings. We performed 2 sensitivity analyses for each outcome; 1) leave-one-out test; 2) variation in covariate selection.

In the leave-one-out tests, we tested whether any single state was disproportionately driving the effect size. We reran the synthetic control model iteratively excluding a single state from the control pool. We then re-estimated the effect sizes from the resulting models and evaluated the outcome. Results are shown in S5 Table of the supplementary materials. Each synthetic control California is comprised of a modest number of states (3 – 5). Our results suggest that for the synthetic control analysis, no single state disproportionately drives our effect size.

In order to evaluate the sensitivity of our outcome to the covariate combinations used, we varied the cut-off for the characteristic covariates (cross validation and covariate selection are described in detail in an earlier section of this appendix). We re-ran the model with varying cutoffs for the characteristic covariates included. We compared the resulting effect sizes. Results are shown in S4 Table. The effect size did not vary strongly with different covariate combinations. As such, we concluded that our model is robust to different characteristic covariate combinations.

**Appendix References**

1. United States Centres for Disease Control and Prevention. VaxView <https://www.cdc.gov/>: United States Centres for Disease Control and Prevention; 2017 [Available from: <https://www.cdc.gov/vaccines/vaxview/index.html>.

2. Abadie A, Gardeazabal J. The Economic Costs of Conflict : A Case Study of the Basque Country. American Economic Review. 2007;93(1):113-32.

3. Abadie A, Diamond A, Hainmueller J. Synthetic Control Methods for Comparative Case Studies: Estimating the Effect of California’s Tobacco Control Program. Journal of the American Statistical Association. 2010;105:493-505.

4. McClelland R, Gault S. The Synthetic Control Method as a Tool to Understand State Policy. 2017.

5. United States Census Bureau. American Community Survey (ACS) <https://www.census.gov/programs-surveys/acs>: United States Census Bureau,; 2019 [Available from: <https://www.census.gov/programs-surveys/acs>.

6. Omer SB, Pan WKY, Halsey NA, Stokley S, Moulton LH, Navar AM, et al. Nonmedical Exemptions to School Immunization Requirements Secular Trends and Association of State Policies With Pertussis Incidence. JAMA. 2006;296(14):1757-63.

7. Hill HA, Elam-Evans LD, Yankey D, Singleton JA, Kang Y. Vaccination Coverage Among Children Aged 19–35 Months — United States, 2016. Morbidity and Mortality Weekly Report. 2017;66(43):1171 - 7.
