# Supplementary material for: The 2016 California policy to eliminate nonmedical vaccine exemptions and changes in vaccine coverage: An empirical policy analysis
Source: PLoS Med. 2019 Dec 23;16(12):e1002994. doi: 10.1371/journal.pmed.1002994 (PMC6927583; doi:10.1371/journal.pmed.1002994)
Supplement: S2 Appendix — (DOCX) [file pmed.1002994.s002.docx]

**S2 Appendix: The Difference-in-Differences Method and County Level Data**

*County Level Data*

County level vaccination and exemption data were individually requested from state health departments of all 50 states and the District of Columbia. At least three separate attempts at contact were made for each state via both phone and email. Our inclusion criteria included: 1) county level data for complete overall vaccination coverage or MMR coverage; 2) a start date of county level data collection between 2010 to 2012 and end date of 2017. Of the 50 states contacted, in addition to California, 16 were able to provide data for overall vaccination coverage and 17 were able to provide data for non-medical and medical exemptions (see S4 Figure). Due to data sharing restrictions, county level data is not included in the study data uploaded to the Figshare Data repository. Interested researchers are encouraged to individually contact the relevant state health departments to request access to the county level vaccination coverage and exemption data. Contact details for the state health departments are listed in the subsequent Table I.

For the states included in the analysis, missing outcomes data was handled in the following manner. First, we excluded individual counties that were missing greater than 25% of data for the specified time period. Second, for the remainder of counties we used simple imputation for missing values.

*Statistical Analysis*

To measure associations of the California policy with vaccination coverage, we compared the change in kindergarten overall vaccination coverage and exemption percentage, at the county level, after the policy’s implementation in 2016 in California counties against the change in control counties. This difference-in-differences strategy removes biases between the treatment and control groups that may exist because of permanent differences between the groups as well as from comparisons over time in the treatment group that may be the result of general trends. The quasi-experimental approach of this model assumes that the policy was introduced at a random time point, and can be exploited as an exogenous variable. An advantage of analyzing the data at the county level is that we could adjust for county-level factors that could affect immunization coverage. Covariate data was obtained from the American Community Survey (ACS) [5]. Based on literature of factors affecting vaccine hesitancy, the following county level variables were pre-specified to be included in the model: average household size, percent white, education (bachelor’s degree or higher, some college or less, high school or less), median income, population size, and children without insurance coverage[6,7]. Race was included as the percent white in a county, as race has been implicated in previous research regarding vaccination coverage [7].

*Sensitivity Analysis*

We performed sensitivity analyses to determine the robustness of our findings. In the leave-one-out test, we reran the model excluding a single state from the control pool. The effect sizes from the resulting models are shown in S9 Table. Our results suggest that no single state disproportionately drives our effect size. As such, our effect size is robust to states included in the analysis. Additionally, given the availability of data from control states, we performed a sub-analysis that excluded states which reported MMR coverage and not overall vaccination coverage. The effect sizes from the resulting model are shown in S10 Table.

**Table I: Contact details for state departments of public health**

| **State** | **Health Department Immunization**  **Division Contact Information** |
| --- | --- |
| Arizona | 602-364-3630 |
| Arkansas | 501-661-2169 |
| Connecticut | 860-509-7929 |
| Florida | 1-877-888-7468 |
| Iowa | 1-800-831-6293 |
| Kansas | 877-296-0464 |
| Maryland | [MDH.IZInfo@maryland.gov](mailto:MDH.IZInfo@maryland.gov) |
| Massachusetts | 617-983-6800 |
| Minnesota | 1-800-657-3970 or 651-201-5503 |
| New Jersey | 609-826-4860 |
| New York | [immunize@health.ny.gov](mailto:immunize@health.ny.gov) |
| North Dakota | 701-328-3386 or 1-800-472-2180 |
| Oregon | imm.info@state.org.us |
| Rhode Island | 401-222-5960 |
| Texas | 903-533-5292 |
| Virginia | 804-864-8055 |
| Washington | 360-236-3595 |

**Appendix References**

1. United States Centres for Disease Control and Prevention. VaxView <https://www.cdc.gov/>: United States Centres for Disease Control and Prevention; 2017 [Available from: <https://www.cdc.gov/vaccines/vaxview/index.html>.

2. Abadie A, Gardeazabal J. The Economic Costs of Conflict : A Case Study of the Basque Country. American Economic Review. 2007;93(1):113-32.

3. Abadie A, Diamond A, Hainmueller J. Synthetic Control Methods for Comparative Case Studies: Estimating the Effect of California’s Tobacco Control Program. Journal of the American Statistical Association. 2010;105:493-505.

4. McClelland R, Gault S. The Synthetic Control Method as a Tool to Understand State Policy. 2017.

5. United States Census Bureau. American Community Survey (ACS) <https://www.census.gov/programs-surveys/acs>: United States Census Bureau,; 2019 [Available from: <https://www.census.gov/programs-surveys/acs>.

6. Omer SB, Pan WKY, Halsey NA, Stokley S, Moulton LH, Navar AM, et al. Nonmedical Exemptions to School Immunization RequirementsSecular Trends and Association of State Policies With Pertussis Incidence. JAMA. 2006;296(14):1757-63.

7. Hill HA, Elam-Evans LD, Yankey D, Singleton JA, Kang Y. Vaccination Coverage Among Children Aged 19–35 Months — United States, 2016. Morbidity and Mortality Weekly Report. 2017;66(43):1171 - 7.
