# Supplementary material for: The 2016 California policy to eliminate nonmedical vaccine exemptions and changes in vaccine coverage: An empirical policy analysis
Source: PLoS Med. 2019 Dec 23;16(12):e1002994. doi: 10.1371/journal.pmed.1002994 (PMC6927583; doi:10.1371/journal.pmed.1002994)
Supplement: S3 Appendix — (DOCX) [file pmed.1002994.s003.docx]

**S3 Appendix: Pre-analysis Plan**

Pre-analysis Plan (A timestamped copy can be found online^12^)

March 11, 2019

**Effectiveness of the 2016 California Policy to Eliminate Non-Medical Exemptions**

**on Vaccine Coverage: A Synthetic Analysis**

Sindiso Nyathi and Nathan C. Lo

Stanford University

**Study Objective**

In July 2016, California Senate Bill 277 (SB277) eliminated personal belief exemptions from immunization for children in childcare and public/private schools.^1^ The goal of this analysis is to examine the relationship between SB277 and vaccination coverage in kindergarteners via a Synthetic Control Analysis.

**Data and Outcomes**

The primary outcome of the study is the state-level vaccine coverage for the MMR vaccine in kindergarteners. We defined two secondary outcomes: 1) percentage of kindergartners with Any Exemption (indicative of the prevalence non-medical or “personal belief” exemptions); and 2) percentage of kindergartners receiving Medical Exemptions.

State level vaccine coverage data for the 2011/12 to 2017/18 school years was retrieved from the CDC’s VaxView website.^8^ SB277 came into effect in the 2016/17 school year. Vaccine coverage data for the 2011/12 to 2014/15 school years was used as pre-policy data, while data for the 2016/17 and 2017/18 school years was used as post-policy data. Covariate data was retrieved from the US Census Bureau and the Data Resource Center for Child and Adolescent Health.^9,10^

**Method**

The Synthetic Control approach was developed by Abadie et al., as an alternative to traditional analytic approaches employed when working with aggregated data in the context of comparative case studies or policy evaluations.^2–4^ The goal of the analysis it to create a ‘synthetic control’ of the treated unit from the non-treated units, known as the donor pool. This synthetic region approximates the behavior of the outcome in the treated region in the absence of the intervention or policy. The synthetic control provides a hypothetical counterfactual for comparison with the actual treated unit, allowing researchers to estimate the magnitude of the treatment effect. The resulting synthetic control is a weighted sum of regions from the donor pool. The weights are chosen to minimize the difference in a set of covariates between the treated unit and the synthetic control in the pre-intervention period. As such, the synthetic control analysis will be conducted in three steps. Step 1 will focus on covariate selection, while step 2 will be the application of the selected covariates to the synthetic control method. The third step is a series of sensitivity tests to assess the robustness of our model.

Possible covariates were identified based on a review of vaccine coverage literature, and expert opinion.^5–7^ The set of covariates to be included in the model was then chosen via a manual stepwise variable inclusion procedure. Each covariate was included stepwise in the model, starting with the covariate that minimized a Root Mean Square Predictive Error (RMSPE) value. Subsequently, covariates that further reduced this value were added to the final set of covariates. The resulting graph did not exhibit a monotonic decrease, and so we chose a set of covariates that minimized RMSPE, with as few terms as possible. We incorporated cross validation into the variable selection procedure to ensure that the resulting variable combination did not overfit the model. To do this we used a subset of the preintervention data to train the model (2011/12 – 2013/14) and the remaining preintervention time period (2014/15 – 2015/16) to test the model.

| **No.** | **Covariates** | **Test error (RMSPE)** |
| --- | --- | --- |
| 1 | No Previous Well child visit (%) | 1.28 |
| 2 | Uninsured (%) | 1.11 |
| 3 | No Consistent Coverage (%) | 1.06 |
| 4 | Private Insurance (%) | 0.83 |
| 5 | Per Capita Health ($) | 0.95 |
| 6 | Population (No.) | 0.95 |
| 7 | Children with No Insurance (%) | 0.78 |
| 8 | Live Rural (%) | 0.79 |
| 9 | Median Income ($) | 1.00 |
| 10 | Married (%) | 1.33 |
| 11 | Bachelor’s Degree or Higher (%) | 1.19 |
| 12 | High School or Higher (%) | 1.22 |
| 13 | Median Age (yrs) | 1.19 |
| 14 | Below Poverty (%) | 1.19 |
| 15 | White (%) | 2.14 |

**Table 1: Test Root Mean Square Predictive Error (RMSPE) values.**

Difference between the vaccine coverage values of the resulting synthetic control and California for the testing years (2014/15 - 2015/16) for a model trained on 2011/12 – 2013/14 data.

**Figure 1.** **Root Mean Square Predictive Error (RMSPE) values.**

The first 4 covariates and the Average Lag value for the preintervention outcome are included as covariates based on where the inflexion points of RMPSE minimization.

Table 1 shows the initial set of covariates suggested for inclusion in the model as well as the RMSPE associated with a model that includes the given variable and every variable prior. Fig. 1 is the corresponding plot and shows the cutoff for inclusion in the model. The variables included in the final model were, 1) No Previous Well Child Visit (%), 2) Uninsured (%), 3) No Consistent Coverage (%), 4) Private Insurance (%). The average of the pre-intervention outcome values (average lag) was included as a covariate following the convention set by Abadie et al. in their analysis. Fig. 2 shows the resulting synthetic control as well as the actual vaccine coverage for 2011/12 to 2015/16.

**Figure 2. Cross Validation.**

Cross Validation was conducted to ensure that the resulting covariate combination did not overfit the model. Pre-intervention data (2011/12 – 2015/16) was divided into training (2011/12, 2013/14) and testing datasets (2014/15, 2015/16).

The 2^nd^ step in the analysis will be to use the chosen covariates to construct a synthetic control. Permutation tests, the primary means of inference used with the synthetic control approach, will be used to detect a meaningful treatment effect. We pre-define the treatment as as “meaningful” if the post-intervention vaccine coverage trajectory is in the top 5% of vaccine coverage trajectories from the permutation tests. Finally, sensitivity analysis will be conducted to test the robustness of the model. The first set of sensitivity analyses will test different combinations of covariates used to create the synthetic control, including variation on the lag variable^11^. The second set of sensitivity tests, ‘Leave one out tests’, exclude a single state from the donor pool to ascertain that no single state is driving the synthetic control.

In addition to the currently presented Synthetic Control analysis, a Difference-in-Differences analysis of the effectiveness of SB277 on vaccine coverage at the country level was conducted using data from a subset of States. A third analysis will combine synthetic control and Difference-in-Differences to identify a relationship. Together the results of these three analyses will provide a comprehensive picture of the effectiveness of California’s SB277 on vaccine coverage at the state and county level.

****UPDATE: Note the authors did not conduct the third analysis (combine synthetic control and difference-in-differences) based on limitations in available data to make this a meaningful analysis.**

**References**

1. Pan, R. & Allen, B. *Senate Bill No. 277*. 1–5 (2018).

2. Abadie, A., Gardeazabal, J., American, T., Review, E. & Mar, N. The Economic Costs of Conflict : A Case Study of the Basque Country The Economic Costs of Conflict : A Case Study of the Basque Country. *Am. Econ. Rev.* **93**, 113–132 (2007).

3. Abadie, A., Diamond, A. & Hainmueller, J. Synthetic Control Methods for Comparative Case Studies: Estimating the Effect of California’s Tobacco Control Program. *J. Am. Stat. Assoc.* **105**, 493–505 (2010).

4. Abadie, A. Comparative Politics and the Synthetic Control Method Alexis Diamond – International Finance Corporation. *Am. J. Pol. Sci.* 1–16 (2012). doi:10.1111/ajps.12116

5. Lu, P. jun *et al.* Association of Health Insurance Status and Vaccination Coverage among Adolescents 13-17 Years of Age. *J. Pediatr.* **195**, 256–262.e1 (2018).

6. Birnbaum, M. S., Jacobs, E. T., Ralston-King, J. & Ernst, K. C. Correlates of high vaccination exemption rates among kindergartens. *Vaccine* **31**, 750–756 (2013).

7. Carpiano, R. M. & Bettinger, J. A. Vaccine coverage for kindergarteners: Factors associated with school and area variation in Vancouver, British Columbia. *Vaccine Reports* **6**, 50–55 (2016).

8. CDC. VaxView | Vaccination Coverage | NIS | Home | CDC. *October 6, 2016* Available at: https://www.cdc.gov/vaccines/vaxview/index.html. (Accessed: 27th November 2018)

9. United States Census Bureau. American FactFinder. Available at: https://factfinder.census.gov/faces/nav/jsf/pages/index.xhtml? (Accessed: 27th November 2018)

10. Data Resource Center for Child and Adolescent Health. (2019). Available at: https://www.childhealthdata.org/. (Accessed: 7th January 2019)

11. Mcclelland, R. & Gault, S. *The Synthetic Control Method as a Tool to Understand State Policy*. (2017).

12. Effectiveness of the 2016 California Policy to Eliminate Non-Medical Exemptions

on Vaccine Coverage: A Synthetic Analysis. (2019). Available at : <https://github.com/NathanLo3/Publication-codes/raw/master/California%20Vaccine%20Coverage%20Analysis-%20synth%20control%20pre-analysis%20plan.pdf>. (Accessed: March 11th 2019).

Pre Analysis Plan

April 2019

**Effectiveness of the 2016 California Vaccine Policy on Vaccine Coverage and Non-Medical Exemptions: A Difference in Difference Analysis**

Hannah Karpel and Nathan C. Lo

Stanford University

**Objective**: In 2016, California implemented a new vaccination policy that eliminates personal belief exemptions from immunization requirements for children in childcare and private schools. The objective of this study is to explore the effectiveness of California’s new vaccine policy in increasing vaccination coverage in kindergarteners using a difference in difference analysis.

**Method:** A difference and difference (DID) model will be used to estimate the relationship between California’s new policy and entry immunization coverage for kindergarteners as well as on medical and nonmedical exemption percentages. DID models have been widely used to conduct policy evaluations^1^. DID uses longitudinal data to estimate the impact of an intervention or treatment, such as the passage of California’s new policy, by comparing the changes in outcome over time between the population subject to the intervention (treatment group) and a population that is not subject to the intervention (control group), before and after the intervention^1^. This model removes biases between the treatment and control groups that may exist because of permanent differences between the groups as well as from comparisons over time in the treatment group that may be the result of general trends^1^. The quasi-experimental approach of this model assumes that the policy was introduced as a random time point, which can be exploited as an exogenous variable. Aggregated county level vaccination data from 2010-2018 for control states (based on availability of data) will be used to estimate the impact of California’s new policy on vaccination coverage. Additional analyses will include a synthetic control model and a combined DID and synthetic control model to examine the impact of California’s new policy in increasing vaccination coverage in kindergarteners.

**Data and Outcomes:**

For the DID model, the primary outcome of the study was defined as county level school entry vaccination coverage in kindergarteners (percentage school entry vaccination coverage). Secondary outcomes were medical and non-medical (“personal belief”) exemptions to vaccine requirements. To collect data for county level vaccine and exemption percentages, we contacted departments of health in all 50 states. We included data for states in which county level data was collected from 2010 through 2018 for both school entry vaccine coverage and medical and non-medical exemptions. When states only reported individual vaccine coverage for counties, we used county level vaccine coverage for the measles-mumps-rubella (MMR) vaccine as indication of kindergarten entry vaccination coverage. We also performed a sub-analysis in which we only analyzed the data from states reporting full entry vaccination coverage. As California’s policy came into effect in the 2016-2017 school year, county vaccine coverage data for the 2010-2011 school year until the 2015-2016 school year (7 years) was used as pre-policy data, and data from the 2016-2017 school year through the 2017-2018 school year was used as post policy data.

Covariate data was retrieved from the American Community Survey (ACS)^2^. This survey is conducted through the U.S. Census Bureau every year for all states and for counties with a population greater than 65,000^3^. Therefore, only counties that were included in both the ACS datasets and the county level vaccine datasets were used in the analysis. The following county level variables were pre-specified to be included in the model: average household size (no), percent white (%), education attainment defined as bachelor’s degree or more (%), some college or less (%), or high school or less (%), median income (no), population size (no), poverty (%), and children without insurance coverage (%)^4-5^.

**Sensitivity Analysis:** Sensitivity analysis will repeat the analysis leaving out one state in turn^6^

**References**

1. “Difference‐in‐Differences Estimation”, Imbens/Wooldridge, Lecture Notes 10, summer 2007. http://www.nber.org/WNE/lect_10_diffindiffs.pdf
2. United States Census Bureau. American FactFinder. Available at: <https://factfinder.census.gov/faces/nav/jsf/pages/index.xhtml>?
3. United States Census Bureau. American Community Survey (ACS). Available at: https://www.census.gov/programs-surveys/acs/data.html
4. Omer SB, Pan WKY, Halsey NA, et al. Nonmedical Exemptions to School Immunization Requirements Secular Trends and Association of State Policies With Pertussis Incidence. JAMA. 2006;296(14):1757–1763. doi:10.1001/jama.296.14.1757
5. Hill HA, Elam-Evans LD, Yankey D, Singleton JA, Kang Y. Vaccination Coverage Among Children Aged 19-35 Months—United States, 2016. MMWR Morb Mortal Wkly Rep 2017;66:1171-1177. Doi:http://dx.doig.org/10.15585/mmwr.mm6643a3
6. Bendavid E, Holmes CB, Bhattacharya J, Miller G. HIV Development Assistance and Adult Mortality in Africa. *JAMA.* 2012;307(19):2060–2067. doi:10.1001/jama.2012.2001
