# Supplementary material for: The 2016 California policy to eliminate nonmedical vaccine exemptions and changes in vaccine coverage: An empirical policy analysis
Source: PLoS Med. 2019 Dec 23;16(12):e1002994. doi: 10.1371/journal.pmed.1002994 (PMC6927583; doi:10.1371/journal.pmed.1002994)
Supplement: S1 Fig — (DOCX) [file pmed.1002994.s005.docx]

**S1 Fig: Characteristic state covariate selection cutoffs**

In the variable selection procedure we used a training (2011, 2012, 2013) and testing set (2014, 2015) to choose the best variable combination. We used stepwise variable selection on the training dataset to create a synthetic control and evaluated the fit using the testing set, and the resulting error calculated as the Root Mean Square Predictive Error (RMSPE) value (S1 Appendix). We iteratively added variables to the model and chose a set of covariates that minimized the RMSPE with as few covariates as possible.
