# Supplementary material for: The 2016 California policy to eliminate nonmedical vaccine exemptions and changes in vaccine coverage: An empirical policy analysis
Source: PLoS Med. 2019 Dec 23;16(12):e1002994. doi: 10.1371/journal.pmed.1002994 (PMC6927583; doi:10.1371/journal.pmed.1002994)
Supplement: S2 Fig — (DOCX) [file pmed.1002994.s006.docx]

**S2 Fig: Cross validation of synthetic controls using training and testing data for variable selection**

For each characteristic covariate combination, we evaluated the model using a cross-validation procedure of the pre-policy data. As described in the Appendix methods, we separated the pre-policy data into training and testing set; we made model predictions for each combination of covariates on the observed data for California for 2014 and 2015. These predictions were used to calculate a Root Mean Square Predictive Error (RMSPE) value to inform variable selection for the final model and to prevent over-fitting the model.
