# Supplementary material for: The 2016 California policy to eliminate nonmedical vaccine exemptions and changes in vaccine coverage: An empirical policy analysis
Source: PLoS Med. 2019 Dec 23;16(12):e1002994. doi: 10.1371/journal.pmed.1002994 (PMC6927583; doi:10.1371/journal.pmed.1002994)
Supplement: S3 Fig — (DOCX) [file pmed.1002994.s007.docx]

**S3 Fig: Placebo plot trajectories for control states (grey) and treated state (blue)**

In placebo tests, we re-assigned treatment status to untreated states and revaluated the difference between the synthetic control and the untreated state. We observe random variation in all states before the treatment. We then computed effect sizes (difference between synthetic control and state) of all states, including the treated state (California) and compared them to determine whether the observed change in outcome for the treated state (California) was meaningful as defined as within the top 5^th^ percentile of changes.
