# Supplementary material for: The 2016 California policy to eliminate nonmedical vaccine exemptions and changes in vaccine coverage: An empirical policy analysis
Source: PLoS Med. 2019 Dec 23;16(12):e1002994. doi: 10.1371/journal.pmed.1002994 (PMC6927583; doi:10.1371/journal.pmed.1002994)
Supplement: S4 Fig — (DOCX) [file pmed.1002994.s008.docx]

**S4 Fig: Flow chart of data collection for county level outcomes from state health departments for control states**

States^a^ contacted by email/phone

(n = 50)

States that responded

(n = 39)

States able to provide data based on inclusion criteria

(n = 35)

States with county level data collection starting in 2010-2012

(n = 16-17)^b^

^a^Included the District of Columbia

^b^Sixteen states were able to provide county level data for vaccination coverage; seventeen states were able to provide data for medical and non-medical exemptions.
