# Supplementary material for: The 2016 California policy to eliminate nonmedical vaccine exemptions and changes in vaccine coverage: An empirical policy analysis
Source: PLoS Med. 2019 Dec 23;16(12):e1002994. doi: 10.1371/journal.pmed.1002994 (PMC6927583; doi:10.1371/journal.pmed.1002994)
Supplement: S5 Fig — (DOCX) [file pmed.1002994.s009.docx]

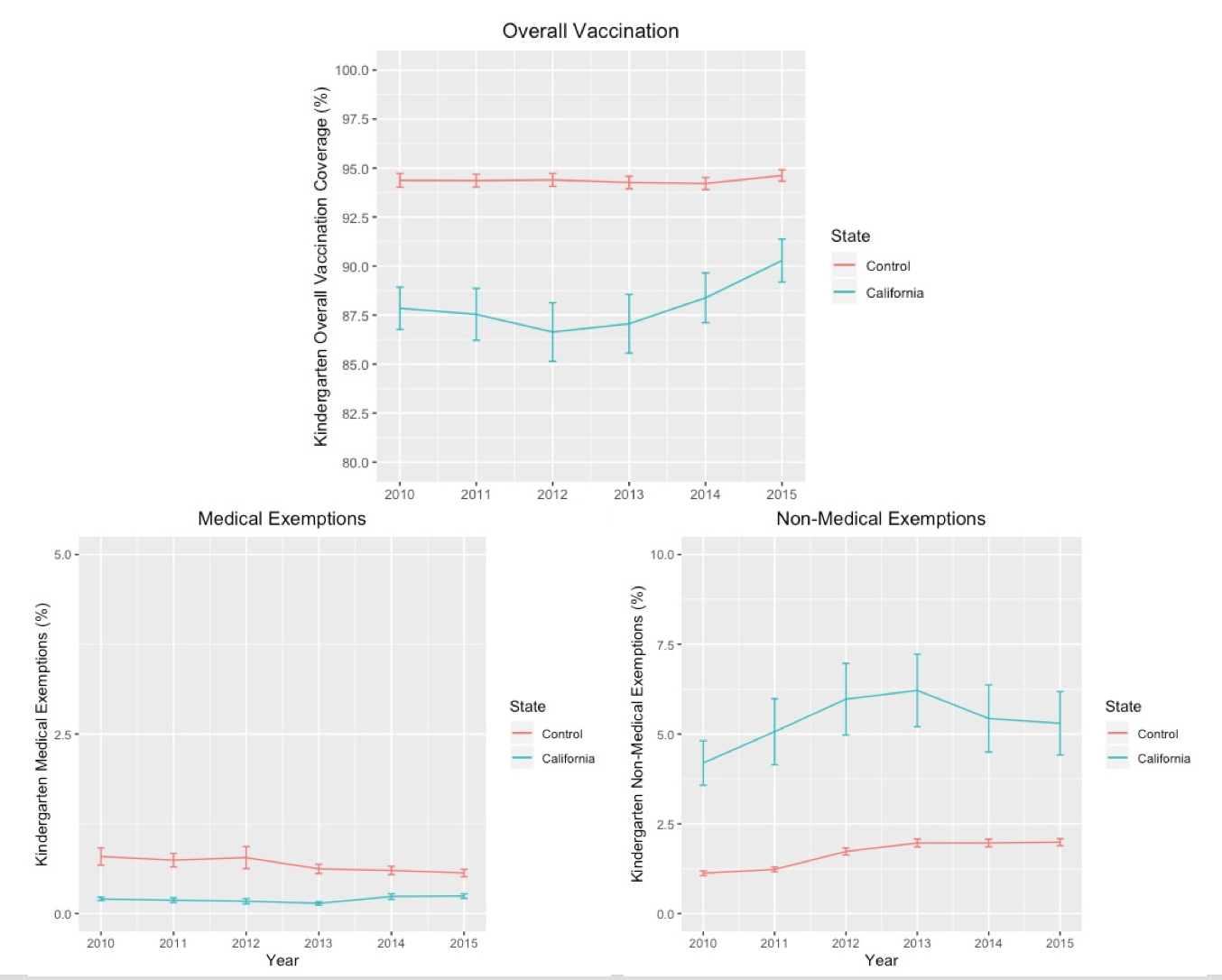


**S5 Fig: Pre-policy trends in outcome variables in California and control counties**

To assess the parallel trends assumption for the county level difference-in-differences analysis, we plotted the average county level vaccination coverage and exemption percentages for California counties and control counties from the 2010-2011 school year through the 2015-2016 school year. The California policy went into effect before the start of the 2016-2017 school year.
