# Supplementary material for: The 2016 California policy to eliminate nonmedical vaccine exemptions and changes in vaccine coverage: An empirical policy analysis
Source: PLoS Med. 2019 Dec 23;16(12):e1002994. doi: 10.1371/journal.pmed.1002994 (PMC6927583; doi:10.1371/journal.pmed.1002994)
Supplement: S1 Table — (DOCX) [file pmed.1002994.s010.docx]

**S1 Table: Data sources for state level synthetic control analysis**

| **Variable** | **Definition** | **Source** |
| --- | --- | --- |
| State level MMR coverage | Proportion of entering kindergartners who received the state-required doses MMR vaccine at the start of the school year | CDC, SchoolVaxView  (<https://www.cdc.gov/vaccines/vaxview/index.html>) |
| State level medical exemptions | Proportion of entering kindergartners who claimed a medical exemption at the start of the school year |
| State level non-medical exemptions | Proportion of entering kindergartners who claimed a non-medical exemption at the start of the school year |
| No previous well child visit (%) | Proportion of children with no visit to a doctor, nurse or other health care professional for preventive medical care such as a physical exam or well-child check-up in past 12 months. | Data Resource Center for Child and Adolescent Health  ([^www.childhealthdata.org^](file:///Users/snyathi/Downloads/Manuscript_materials_final_issues/www.childhealthdata.org)) |
| No consistent coverage (%) | Proportion of children with persistent health insurance coverage in last 12 months |
| Private insurance (%) | Proportion of children with private insurance at the time of the interview |
| Children with no insurance (%) | Proportion of children with no health care coverage (health insurance, HMOs, government plans etc.) |
| Per capita health ($) | Health spending per capita | Centers for Medicare and Medicaid Services  (<https://www.cms.gov/2017>) |
| Live rural (%) | Proportion of children living in rural areas of the United States | United States Census Bureau  (<https://www.census.gov/programs-surveys/acs>) |
| Median income ($) | Median income |
| Married (%) | Married at time of interview |
| Bachelor’s degree or higher (%) | Proportion with bachelor’s degree or higher |
| High school or higher (%) | Proportion with high school diploma or higher |
| Median age (yrs) | Median age |
| Below poverty (%) | Proportion of families and people whose income in the past 12 months was below the poverty level. |
| Uninsured (%) | Proportion uninsured (including adults) |
| Population (No.) | Population |
| White (%) | Proportion white |

Abbreviations: MMR, Measles Mumps and Rubella Vaccine; HMO, Health Maintenance Organization

Publicly available state level MMR coverage and medical, and non-medical exemption data, as well as demographic and health related data were retrieved from various sources, as listed in the table
