# Supplementary material for: The 2016 California policy to eliminate nonmedical vaccine exemptions and changes in vaccine coverage: An empirical policy analysis
Source: PLoS Med. 2019 Dec 23;16(12):e1002994. doi: 10.1371/journal.pmed.1002994 (PMC6927583; doi:10.1371/journal.pmed.1002994)
Supplement: S2 Table — (DOCX) [file pmed.1002994.s011.docx]

**S2 Table: States excluded from control pool due to missing data**

| **MMR coverage**  **(43 States included)** | **Non-medical exemptions**  **(43 States included)** | **Medical exemptions**  **(44 States included)** |
| --- | --- | --- |
| Alaska  District of Columbia  Hawaii  New Hampshire  New Jersey  North Carolina  Wyoming  Oklahoma | Colorado  Illinois  Minnesota  Mississippi  Missouri  West Virginia  Wyoming  South Carolina | Colorado  Illinois  Minnesota  Missouri  Texas  Wyoming  North Carolina |

Abbreviations: MMR, Measles Mumps and Rubella Vaccine

For each outcome, the synthetic control analysis excluded a control state from the pool of potential controls if data for the given outcome was unavailable for at least 1 year.
