# Supplementary material for: The 2016 California policy to eliminate nonmedical vaccine exemptions and changes in vaccine coverage: An empirical policy analysis
Source: PLoS Med. 2019 Dec 23;16(12):e1002994. doi: 10.1371/journal.pmed.1002994 (PMC6927583; doi:10.1371/journal.pmed.1002994)
Supplement: S3 Table — (DOCX) [file pmed.1002994.s012.docx]

**S3 Table: Characteristic covariate weights used to create the synthetic California for each outcome in base case analysis**

| **MMR Coverage** | | **Non-medical Exemptions** | | **Medical Exemptions** | |
| --- | --- | --- | --- | --- | --- |
| **Covariate** | **Weight**  **(%)** | **Covariate** | **Weight**  **(%)** | **Covariate** | **Weight**  **(%)** |
| Average MMR Lag | 96 | Average MMR Lag | 90 | Average MMR Lag | 98 |
| Median Age | 0.01 | Median Age | 8.7 | Private Insurance | 2 |
| No Well Child Visit | 2.6 | Population | 0.4 | Per Capital Health | 0 |
| Uninsured | 0.7 | Education, Bachelor’s degree | 0.4 |  |  |
| Private Insurance | 1.2 | No Coverage | 0.07 |  |  |
| Population | 0.9 |  |  |  |  |

Abbreviations: MMR, Measles Mumps and Rubella Vaccine.

The synthetic control method creates a synthetic control California that matches the treated California on the pre-policy outcome and on a set of weighted characteristic covariates. The respective weights for the characteristic covariates shown suggest that the average pre-policy lag variable consistently has the greatest influence on the resulting synthetic control.
