# Supplementary material for: The 2016 California policy to eliminate nonmedical vaccine exemptions and changes in vaccine coverage: An empirical policy analysis
Source: PLoS Med. 2019 Dec 23;16(12):e1002994. doi: 10.1371/journal.pmed.1002994 (PMC6927583; doi:10.1371/journal.pmed.1002994)
Supplement: S4 Table — (DOCX) [file pmed.1002994.s013.docx]

**S4 Table: Characteristic covariate sensitivity analysis for state level synthetic control analysis**

| **Covariate** | **MMR Coverage** | **Covariate** | **Non-medical Exemptions** | **Covariate** | **Medical Exemptions** |
| --- | --- | --- | --- | --- | --- |
| Average pre-policy lag | 3.23 | Average pre-policy lag | -2.47 | Average pre-policy lag | 0.39 |
| Median Age | 3.34 | Median Age | -2.64 | Private Insurance | 0.39 |
| No Well Child Visit | 3.47 | Population | -2.37 | Per Capita health expenditure | 0.39 |
| Uninsured | 3.62 | Education, Bachelor’s degree | -2.78 | Median Age | 0.40 |
| Private Insurance | 3.34 | No Coverage | -2.36 | Median Income | 0.40 |
| Population | 3.18 | Live Rural | -2.37 | Children with no Insurance | 0.35 |
| Per Capita health expenditure | 3.49 | Per Capita health expenditure | -2.42 | No Previous Well Child Visit | 0.38 |
| Children with no Insurance | 3.13 | Children with no Insurance | -2.49 | No Coverage | 0.36 |
| Median Income | 3.40 | Below Poverty | -2.22 | Married | 0.38 |
| All Covariates | 3.23 | All Covariates | -2.36 | All Covariates | 0.36 |
| **Reported [Range]** | **3.34**  **[3.13 - 3.62]** |  | **-2.36**  **[2.78-2.22]** |  | **0.39**  **[0.35 - 0.40]** |

Abbreviations: MMR, Measles Mumps and Rubella Vaccine.

We varied the characteristic covariate combination used to create the synthetic control in order to evaluate the influence of the covariates on the effect size. To do this we varied the cutoff for covariate inclusion in the model and re-evaluated the effect size (e.g. for MMR coverage we re-ran the model with just average pre-policy lag, then reran the model with average pre-policy lag + median age, then re-ran the model with lag + median age + well child visits, etc., until we had a model with all covariates). The resulting range of effect sizes suggests that our model is robust to characteristic covariate combinations.
