# Supplementary material for: The 2016 California policy to eliminate nonmedical vaccine exemptions and changes in vaccine coverage: An empirical policy analysis
Source: PLoS Med. 2019 Dec 23;16(12):e1002994. doi: 10.1371/journal.pmed.1002994 (PMC6927583; doi:10.1371/journal.pmed.1002994)
Supplement: S5 Table — (DOCX) [file pmed.1002994.s014.docx]

**S5 Table: Control state sensitivity analysis with leave-one-out tests**

|  | **Unit Excluded** | **MMR** | **Non-medical** | **Medical** |
| --- | --- | --- | --- | --- |
| 1 | None | 3.34 | -2.36 | 0.39 |
| 2 | Alabama | 3.41 | -2.36 | 0.40 |
| 3 | Alaska | 3.34 | -2.36 | 0.40 |
| 4 | Arizona | 3.28 | -2.36 | 0.41 |
| 5 | Arkansas | 3.74 | -2.36 | 0.40 |
| 6 | Colorado | 3.27 | -2.36 | 0.39 |
| 7 | Connecticut | 3.33 | -1.92 | 0.40 |
| 8 | Delaware | 3.26 | -2.36 | 0.40 |
| 9 | District of Columbia | 3.34 | -2.36 | 0.40 |
| 10 | Florida | 3.21 | -2.37 | 0.40 |
| 11 | Georgia | 3.23 | -2.27 | 0.39 |
| 12 | Hawaii | 3.34 | -2.37 | 0.40 |
| 13 | Idaho | 4.04 | -2.36 | 0.40 |
| 14 | Illinois | 3.19 | -2.36 | 0.39 |
| 15 | Indiana | 3.21 | -2.36 | 0.39 |
| 16 | Iowa | 3.31 | -2.36 | 0.40 |
| 17 | Kansas | 3.24 | -2.36 | 0.40 |
| 18 | Kentucky | 3.68 | -2.36 | 0.40 |
| 19 | Louisiana | 3.31 | -2.36 | 0.40 |
| 20 | Maine | 3.30 | -2.36 | 0.40 |
| 21 | Maryland | 3.26 | nd | 0.40 |
| 22 | Massachusetts | 3.25 | -2.36 | 0.39 |
| 23 | Michigan | 3.30 | -2.68 | 0.40 |
| 24 | Minnesota | 3.26 | -2.36 | 0.39 |
| 25 | Mississippi | 3.55 | -2.36 | 0.39 |
| 26 | Missouri | 3.25 | -2.36 | 0.39 |
| 27 | Montana | 3.24 | -2.37 | 0.40 |
| 28 | Nebraska | 3.34 | -2.36 | 0.40 |
| 29 | Nevada | 3.26 | -2.37 | 0.40 |
| 30 | New Hampshire | 3.34 | -2.36 | 0.40 |
| 31 | New Jersey | 3.34 | -2.35 | 0.40 |
| 32 | New Mexico | 3.18 | -2.36 | 0.40 |
| 33 | New York | 3.41 | -2.68 | 0.40 |
| 34 | North Carolina | 3.34 | nd | 0.40 |
| 35 | North Dakota | 3.32 | -2.77 | 0.40 |
| 36 | Ohio | 3.23 | -2.36 | 0.40 |
| 37 | Oklahoma | 3.34 | -2.36 | 0.40 |
| 38 | Oregon | 3.31 | -1.99 | 0.40 |
| 39 | Pennsylvania | 3.21 | -2.36 | 0.40 |
| 40 | Rhode Island | 3.48 | -2.36 | 0.39 |
| 41 | South Carolina | 3.42 | -2.36 | 0.39 |
| 42 | South Dakota | 3.28 | -2.36 | 0.40 |
| 43 | Tennessee | 3.22 | -2.36 | 0.40 |
| 44 | Texas | 3.51 | -2.23 | 0.39 |
| 45 | Utah | 3.30 | -2.68 | 0.41 |
| 46 | Vermont | 3.23 | -2.38 | 0.40 |
| 47 | Virginia | 3.23 | -2.36 | 0.40 |
| 48 | Washington | 3.26 | -2.37 | 0.40 |
| 49 | West Virginia | 3.23 | -2.36 | 0.40 |
| 50 | Wisconsin | 3.25 | -2.37 | 0.40 |
| **Reported Value [Range]** | | 3.34  [3.18 – 4.04] | -2.36  [1.92 - 2.77] | 0.39  [0.39 – 0.41] |

Abbreviations: MMR, Measles Mumps and Rubella Vaccine; nd, no data due to singular matrices

We evaluated the influence of states included in the control pool to ensure that no single state had a disproportionate influence on the effect size and that slight perturbations of state weights did not significantly change the effect size. We iteratively re-ran the model, excluding a single state from the control pool, and re-evaluated the effect size. The resulting range of effect sizes suggests that even with slightly different control pools, and therefore slightly different weights, the effect size remains stable. In addition, no single state was driving the effect size.
