# Supplementary material for: The 2016 California policy to eliminate nonmedical vaccine exemptions and changes in vaccine coverage: An empirical policy analysis
Source: PLoS Med. 2019 Dec 23;16(12):e1002994. doi: 10.1371/journal.pmed.1002994 (PMC6927583; doi:10.1371/journal.pmed.1002994)
Supplement: S6 Table — (DOCX) [file pmed.1002994.s015.docx]

**S6 Table: List of control states for the county level difference-in-differences analysis for each outcome**

| **Overall vaccination** | **Medical exemptions** | **Non-medical exemptions** |
| --- | --- | --- |
| Arizona | Arizona | Arizona |
| Kansas | Kansas | Kansas |
| Florida | Florida | Florida |
| Iowa | Iowa | Iowa |
| Texas | Texas | Texas |
| Massachusetts | Massachusetts | Massachusetts |
| Maryland | Maryland | Maryland |
| New Jersey | New Jersey | New Jersey |
| Minnesota | Minnesota | Minnesota |
| New York | New York | New York |
| North Dakota | North Dakota | North Dakota |
| Virginia | Virginia | Virginia |
| Rhode Island | Rhode Island | Rhode Island |
| Connecticut | Connecticut | Connecticut |
| Washington | Washington | Washington |
| Oregon | Oregon | Oregon |
| -- | Arkansas | Arkansas |

Inclusion was dependent on the availability of county level data from 2010-2017 from state health departments for each outcome.
