# Supplementary material for: The 2016 California policy to eliminate nonmedical vaccine exemptions and changes in vaccine coverage: An empirical policy analysis
Source: PLoS Med. 2019 Dec 23;16(12):e1002994. doi: 10.1371/journal.pmed.1002994 (PMC6927583; doi:10.1371/journal.pmed.1002994)
Supplement: S7 Table — (DOCX) [file pmed.1002994.s016.docx]

**S7 Table: Pre-Policy trends for outcome variables in California and control states**

| **Year** | **Average Overall Vaccination Coverage (California)** | **Average Overall Vaccination Coverage (Control States)** | **Average Medical Exemptions (California)** | **Average Medical Exemptions (Control States)** | **Average Non-Medical Exemptions (California)** | **Average Non-Medical Exemptions (Control States)** |
| --- | --- | --- | --- | --- | --- | --- |
| 2010 | 87.8% | 94.4% | 0.2% | 0.8% | 4.2% | 1.1% |
| 2011 | 87.5% | 94.4% | 0.2% | 0.7% | 5.1% | 1.2% |
| 2012 | 86.6% | 94.4% | 0.2% | 0.8% | 6.0% | 1.7% |
| 2013 | 87.1% | 94.3% | 0.1% | 0.6% | 6.2% | 2.0% |
| 2014 | 88.4% | 94.2% | 0.2% | 0.6% | 5.4% | 2.0% |
| 2015 | 90.3% | 94.6% | 0.2% | 0.6% | 5.3% | 2.0% |

To evaluate similarity between California and control states in pre-policy trends, we calculated average county level vaccination coverage and exemption percentages provided by state health departments for the pre-policy period.
