# Supplementary material for: The 2016 California policy to eliminate nonmedical vaccine exemptions and changes in vaccine coverage: An empirical policy analysis
Source: PLoS Med. 2019 Dec 23;16(12):e1002994. doi: 10.1371/journal.pmed.1002994 (PMC6927583; doi:10.1371/journal.pmed.1002994)
Supplement: S8 Table — (DOCX) [file pmed.1002994.s017.docx]

**S8 Table: Change in county level outcome variables between 2015 and 2017 for California counties**

| **County** | **Change in Overall Vaccination Coverage 2015-2017 (%)** | **Change in Medical Exemptions 2015-2017 (%)** | **Change in Non-Medical Exemptions 2015-2017 (%)** |
| --- | --- | --- | --- |
| Alameda | 0.5 | 2.0 | -0.1 |
| Amador | 4.4 | 0.5 | -2.5 |
| Butte | 4.1 | 2.1 | -5.9 |
| Calaveras | -2.9 | 3.8 | -11.2 |
| Colusa | -2.5 | 0.8 | 0.8 |
| Contra Costa | 0.7 | 1.9 | -0.7 |
| Del Norte | 8.8 | 3.4 | -6.9 |
| Eldorado | 1.7 | 2.6 | -8.6 |
| Fresno | 0.0 | 1.5 | -0.1 |
| Glenn | 2.4 | 3.0 | 1.0 |
| Humboldt | 8.1 | 6.7 | -12.5 |
| Imperial | 2.9 | 1.4 | -0.1 |
| Inyo | -2.7 | 3.0 | 2.8 |
| Kern | 0.4 | 1.1 | -1.2 |
| Kings | 1.3 | 1.7 | -0.6 |
| Lake | 4.0 | 1.4 | -3.4 |
| Lassen | 14.7 | 1.0 | -10.8 |
| Los Angeles | 5.1 | 1.6 | -0.8 |
| Madera | 1.2 | 0.8 | -2.6 |
| Marin | 4.5 | 3.5 | -5.8 |
| Mariposa | 6.8 | 1.7 | -10.7 |
| Mendocino | 3.8 | 4.6 | -9.6 |
| Merced | -1.0 | 0.8 | -0.3 |
| Modoc | 9.3 | 3.5 | 2.1 |
| Mono | -5.6 | 3.0 | -5.9 |
| Monterey | 2.9 | 1.8 | -1.0 |
| Napa | 3.4 | 2.8 | -2.9 |
| Nevada | 4.1 | 8.0 | -21.5 |
| Orange | 3.4 | 1.8 | -2.7 |
| Placer | 3.3 | 3.1 | -6.4 |
| Plumas | 0.0 | 9.3 | -9.9 |
| Riverside | 1.6 | 1.0 | -1.5 |
| Sacramento | 6.0 | 2.0 | -3.4 |
| San Benito | 1.1 | 1.9 | 0.9 |
| San Bernardino | 1.1 | 1.2 | -0.8 |
| San Diego | 1.6 | 2.1 | -3.0 |
| San Francisco | 1.8 | 2.7 | 0.4 |
| San Joaquin | -0.2 | 1.9 | -0.3 |
| San Luis Obispo | 4.6 | 2.8 | -4.5 |
| San Mateo | 0.5 | 2.3 | 0.2 |
| Santa Barbara | 3.0 | 2.5 | -4.8 |
| Santa Clara | 1.6 | 2.0 | -0.2 |
| Santacruz | 8.4 | 3.9 | -11.1 |
| Shasta | 7.4 | 2.5 | -10.4 |
| Sierra | -6.0 | 3.0 | 0 |
| Siskiyou | 5.5 | 3.0 | -6.1 |
| Solano | 1.3 | 1.7 | 0.0 |
| Sonoma | 0.8 | 4.4 | -5.9 |
| Stanislaus | 0.4 | 2.0 | -0.9 |
| Sutter | -4.6 | 1.5 | -7.5 |
| Tehama | 1.4 | 2.2 | -1.5 |
| Trinity | 26.2 | 5.0 | -12.8 |
| Tulare | -0.4 | 1.8 | 0.3 |
| Tuolumne | 11.2 | 1.4 | -9.4 |
| Ventura | 2.5 | 2.2 | -2.7 |
| Yolo | 1.5 | 2.3 | -3.6 |
| Yuba | -2.0 | 0.7 | -1.8 |
