# Supplementary material for: The 2016 California policy to eliminate nonmedical vaccine exemptions and changes in vaccine coverage: An empirical policy analysis
Source: PLoS Med. 2019 Dec 23;16(12):e1002994. doi: 10.1371/journal.pmed.1002994 (PMC6927583; doi:10.1371/journal.pmed.1002994)
Supplement: S9 Table — (DOCX) [file pmed.1002994.s018.docx]

**S9 Table: County level sensitivity analysis with leave-one-out tests**

| **Excluded State** | **Overall Vaccination Coverage** | **Medical Exemptions** | **Non-Medical Exemptions** |
| --- | --- | --- | --- |
| North Dakota | 4.38 (2.93 - 5.83) | 2.44 (1.99-2.89) | -3.92 (5.41-2.43) |
| New York | 4.36 (2.90- 5.81) | 2.44 (1.98-2.90) | -4.00 (5.49-2.51) |
| Minnesota | 4.28 (2.82 - 5.73) | 2.45 (2.00-2.90) | -3.91 (5.40-2.42) |
| New Jersey | 4.34 (2.88 - 5.80) | 2.45 (2.00-2.90) | -3.93 (5.52-2.44) |
| Rhode Island | 4.36 (2.91 - 5.81) | 2.44 (1.99-2.89) | -3.95 (5.44-2.46) |
| Oregon | 4.41 (2.95 - 5.87) | 2.44 (1.99-2.89) | -3.90 (5.38-2.42) |
| Maryland | 4.46 (3.01 - 5.91) | 2.47 (2.02-2.92) | -3.96 (5.45-2.47) |
| Massachusetts | 4.39 (2.94 - 5.82) | 2.44 (1.99-2.89) | -3.96 (5.45-2.47) |
| Texas | 3.90 (2.42 - 5.38) | 2.65 (2.19-3.11) | -3.93 (5.44-2.42) |
| Virginia | 4.23 (2.76 - 5.70) | 2.45 (2.00-2.90) | -3.94 (5.43-2.45) |
| Iowa | 4.27 (2.81 - 5.73) | 2.44 (1.99-2.89) | -3.94 (5.43-2.45) |
| Florida | 4.52 (3.06 - 5.98) | 2.16 (1.71-2.61) | -3.82 (5.29-2.35) |
| Connecticut | 4.34 (2.88 - 5.80) | 2.45 (2.00-2.90) | -3.94 (5.43-2.45) |
| Kansas | 4.38 (2.93 - 5.83) | 2.45 (2.00-2.90) | -3.93 (5.42-2.44) |
| Washington | 4.26 (2.83 - 5.69) | 2.47 (2.02-2.92) | -3.86 (5.35-2.37) |
| Arizona | 4.34 (2.89 - 5.79) | 2.48 (2.03-2.93) | -4.00 (5.49-2.51) |
| Arkansas | __ | 2.44 (1.99-2.89) | -3.95 (5.44-2.46) |

We evaluated the influence of states included in the control pool for the county level difference-in-differences analysis to ensure that no single state had a disproportionate influence on the effect size. We iteratively re-ran the model, excluding a single state from the control pool, and reevaluated the effect size. The resulting range of effect sizes suggests that no single state was driving the effect size.
