# Supplementary material for: The 2016 California policy to eliminate nonmedical vaccine exemptions and changes in vaccine coverage: An empirical policy analysis
Source: PLoS Med. 2019 Dec 23;16(12):e1002994. doi: 10.1371/journal.pmed.1002994 (PMC6927583; doi:10.1371/journal.pmed.1002994)
Supplement: S10 Table — (DOCX) [file pmed.1002994.s019.docx]

**S10 Table: County level sensitivity analysis with subset of data reporting overall vaccine coverage**

| **Parameter** | **Overall vaccination coverage (95% CI)^b^** | **Medical exemptions prevalence (95% CI)^b^** | **Non-medical exemptions prevalence (95% CI)^b^** |
| --- | --- | --- | --- |
| **2016 California policy ^a^** | 4.1 (2.6 – 5.6) | 2.7 (2.2 – 3.2) | -3.9 (5.4 - 2.4) |
| **Median income per $10,000 (no)** | 1.2 (0.6 – 1.8) | -0.3 (0.6 - 0.1) | -0.2 (0.5 – 0.001) |
| **Mean household size (no)** | -1.9 (-5.0 – 1.2) | 1.2 (0.1 – 2.5) | -0.1 (-1.3 – 1.1) |
| **Population per 100,000 (no)** | 0.008 (-0.09 – 0.1) | 0.05 (-0.02 – 0.1) | 0.002 (-0.5 - 0.5) |
| **Poverty per 1,000 (%)** | 0.7 (-4.2 – 5.7) | -0.04 (-1.3 - 1.2) | -1.1 (-2.9 – 0.7) |
| **White (%)** | -0.02 (-0.08 – 0.03) | 0.02 (-0.003 - 0.05) | 0.03 (0.003 - 0.05) |
| **Education: Less than high school (%)** | 0.4 (0.1 - 0.7) | -0.07 (0.1 - 0.02) | -0.4 (0.5 - 0.2) |
| **Education: Some college or less (%)** | 0.09 (-0.2 - 0.4) | -0.02 (-0.1 – 0.05) | -0.2 (0.3 - 0.08) |
| **Education: Bachelor's Degree or higher (%)** | -0.03 (-0.2 - 0.3) | 0.02 (-0.05 – 0.09) | -0.2 (0.1 - 0.3) |
| **Uninsured children (%)** | -0.1 (-0.2 - 0.02) | 0.1 (0.08 - 0.2) | 0.1 (0.06 - 0.2) |

Abbreviations: CI, Confidence Interval

^a^Difference-in-differences estimates represent relative change in county level vaccination and exemption prevalence for kindergartners in California before and after the 2016 policy

^b^Robust standard errors, clustered by county

Note: We reran the difference-in-differences regression using only states who reported overall vaccination coverage. Five states (North Dakota, Rhode Island, Texas, Maryland, and Minnesota) reported MMR coverage only and were excluded. The results suggest using MMR coverage as a proxy for overall coverage for states who did not report overall coverage did not change the effect sizes for our outcome variables.
