# Supplementary material for: The 2016 California policy to eliminate nonmedical vaccine exemptions and changes in vaccine coverage: An empirical policy analysis
Source: PLoS Med. 2019 Dec 23;16(12):e1002994. doi: 10.1371/journal.pmed.1002994 (PMC6927583; doi:10.1371/journal.pmed.1002994)
Supplement: Attachment — Submitted filename: nyathi.pdf [file pmed.1002994.s020.pdf]

This paper uses routine data-sets to evaluate the impact of a policy change on vaccination coverage in California.

I was asked for a statistical report and I interpret that to include all aspects of the design and conduct of the study.

Points of detail

Page 6 I wonder whether, for an international audience, we need an explanation of what a kindergarten is in the United States context and how it fits into the overall educational system? For instance are they compulsory? Are they the sole gateway to later education?

Page 6 One analysis uses MMR coverage, the other uses overall coverage. There is probably a good reason for this but we should be told. Did the policy change only affect medical exemptions for MMR? If overall vaccination coverage is important what exactly does it cover over and above MMR?

Page 7 If placebo testing is the accepted phrase in the synthetic control world then fine but otherwise it seems a misnomer to me.

Page 9 It might be better to choose a repository which issues a DOI or one belonging to a university. I understand GitHub has quite a steep learning curve.

Page 16, Figure 4 Since the x–axis variable is presumably negatively correlated with part of the composite y–axis variable is there some artefact here as well as any possible true effect?

References Some of these could use checking. Reference 31 looks rather incomplete for example.

I see from that invaluable resource the internet that kindergartner is used in the US for children attending a kindergarten. In some places the authors use the phrase children attending kindergarten which might be preferable to help those German speakers who use Kindergärtner(in) to mean an employee at a Kindergarten

Points of more substance

The synthetic control model

I am not familiar with synthetic control models, an ignorance which I suspect I might share with many readers of the journal. I think a few more details might help. The description in the appendix leaves open the question of where the weights come from. Are these coefficients from the regressions? That seems very unlikely so where do they come from?

If one wanted to be very unkind one might state that the authors have not used a synthetic California but three very different synthetic Californias, one for each outcome. Given that the outcomes are closely related since they sum to unity this seems strange. Would the authors like to explain this further?

The authors have undertaken extensive regression analyses to arrive at their models. This means the coefficients will be very data-driven. This may or may not matter since the goal is prediction not explanation but I do question how stable the weights are. Would it make much difference if equal weights were used especially for the MMR coverage data? There is an interesting discussion of the use of equal coefficients in Wainer (1976).

County model

The differences in differences analysis relies on the parallel trend assumption. This is untestable ofc ourse but is there any evidence that the tends were parallel before the intervention? In the Appendix the authors state 'As with the state level analysis, our results suggest that no single state disproportionately drives our effect size.' The bottom two panels in Figure 2 suggest to me that the first six words might be removed from the sentence. I agree it is not just one state driving things there but it is only a handful.

Summary

Points for clarification and more explanation of the modelling. The fact that the two different methods give broadly similar results increases credibility.

Michael Dewey

References

H Wainer. Estimating coefficients in linear models: it don't make no nevermind. *Psychological Bulletin*, 83:213–217, 1976.
