# Supplementary material for: The 2016 California policy to eliminate nonmedical vaccine exemptions and changes in vaccine coverage: An empirical policy analysis
Source: PLoS Med. 2019 Dec 23;16(12):e1002994. doi: 10.1371/journal.pmed.1002994 (PMC6927583; doi:10.1371/journal.pmed.1002994)
Supplement: Attachment — Submitted filename: Submission 2. Response to Editor.docx [file pmed.1002994.s021.docx]

“Impact of the 2016 California Policy to Eliminate Non-Medical Exemptions on Vaccine Coverage: An Empirical Policy Analysis” (your reference no. PMEDICINE-D-19-02566) – Point-by-point response

29 September 2019

Dear Editor,

We are pleased to submit a revised version of our Original Research article, entitled “Impact of the 2016 California Policy to Eliminate Non-Medical Exemptions on Vaccine Coverage: An Empirical Policy Analysis” (your reference no. PMEDICINE-D-19-02566). We thank you and the three external reviewers very much for the careful examination and helpful comments, and for the invitation to submit a revised manuscript. We have addressed the issues brought forward. In particular, we now present a clearer explanation of the synthetic control analysis, especially for those who may be unfamiliar with the methodology, as well as address additional methodological details. Below, please find our point-by-point response, clearly indicating the changes made in the manuscript.

We believe this article is timely given the increasing public health challenge of vaccine hesitancy and vaccine-preventable disease outbreaks. We trust that our findings will be of interest to the readership of PLOS Medicine, and will support the political debate of state level vaccine policy across the United States.

Sincerely,

Nathan C. Lo, MD PhD

University of California, San Francisco

On behalf of co-authors

**Response to Editor**

*Comment #1*
Did your study have a prospective protocol or analysis plan? Please state this (either way) early in the Methods section.

a) If a prospective analysis plan (from your funding proposal, IRB or other ethics committee submission, study protocol, or other planning document written before analyzing the data) was used in designing the study, please include the relevant prospectively written document with your revised manuscript as a Supporting Information file to be published alongside your study, and cite it in the Methods section. A legend for this file should be included at the end of your manuscript.

Response: We appreciate the editor’s comment on the value of a pre-analysis plan. Our study did have a prospective analysis plan. The document was approved by authors prior to the analysis. We have clarified this point in the Methods section. We have also added the full pre-analysis plan to the S3 Appendix section of the Supplementary materials, as well as a link to the online version.

-In Methods

**Our study objectives, methods, and planned analyses were pre-specified in a pre-analysis plan (S3 Appendix) [34].** This study is reported as per the Strengthening the Reporting of Observational Studies in Epidemiology (STROBE) guideline (S4 Appendix). All statistical analyses were conducted in R (Version 3.5.1). Public datasets and analytical materials are available online (see Appendix) [35, 36]. This was not human subjects research as the study relied on publicly available, aggregated data and was exempt from Institutional Review Board approval.

b) If no such document exists, please make sure that the Methods section transparently describes when analyses were planned, and when/why any data-driven changes to analyses took place.

c) In either case, changes in the analysis—including those made in response to peer review comments—should be identified as such in the Methods section of the paper, with rationale.

Response: The current primary analysis and outcomes are consistent with the pre-analysis plan. We have ensured the Methods section reflects this.

*Comment #2*
2. Thank you for agreeing to make your data available. The provided Github link (from Reference 29) to the dataset does not seem to work. At this time, please provide the corrected link to the data repository and accession numbers required for access.

Response: We have reviewed the relevant data sharing policies for PLOS Medicine. We have uploaded all publicly available data (state level vaccine coverage, state level exemption data) to a Figshare data repository as per recommended PLOS guidelines. We have updated the reference section with the repository link. Based on data sharing agreements with the state Departments of Public Health, we are unable to make some county level vaccine coverage and exemption data available based on restrictions set by states. To address this, we have added a section in the S2 Appendix of the Supplementary Materials clarifying how researchers can get access to this data to facilitate data sharing.

-In S2 Appendix:

The Differences-in-difference method and county level data.

County level vaccination and exemption data were individually requested from state health departments of all 50 states and the District of Columbia. At least three separate attempts at contact were made for each state via both phone and email. Our inclusion criteria included: 1) county level data for complete overall vaccination coverage or MMR coverage; 2) a start date of county level data collection between 2010 to 2012 and end date of 2017. Of the 50 states contacted, in addition to California, 16 were able to provide data for overall vaccination coverage and 17 were able to provide data for non-medical and medical exemptions (see S4 Figure). **Due to data sharing restrictions, county level data is not included in the study data uploaded to the Figshare Data repository. Interested researchers are encouraged to individually contact the relevant state health departments to request access to the county level vaccination coverage and exemption data. Contact details for the state health departments are listed in the subsequent table.**

**Table I: Contact details for state departments of public health**

| **State** | **Health Department Immunization Division Contact Information** |
| --- | --- |
| Arizona | [602-364-3630](tel://6023643630/) |
| Arkansas | 501-661-2169 |
| Connecticut | 860-509-7929 |
| Florida | 1-877-888-7468 |
| Iowa | 1-800-831-6293 |
| Kansas | 877-296-0464 |
| Maryland | [MDH.IZInfo@maryland.gov](mailto:MDH.IZInfo@maryland.gov) |
| Massachusetts | [(617) 983-6800.](tel:6179836800) |
| Minnesota | 1-800-657-3970 or 651-201-5503 |
| New Jersey | 609-826-4860 |
| New York | [immunize@health.ny.gov](mailto:immunize@health.ny.gov) |
| North Dakota | 701-328-3386 or 1-800-472-2180 |
| Oregon | [imm.info@state.or.us](mailto:imm.info@state.or.us) |
| Rhode Island | Tricia.Washburn@health.ri.gov |
| Texas | 903-533-5292 |
| Virginia | 804-864-8055 |
| Washington | 360-236-3595 |

-In References:

34.	Nyathi S, Nathan L. Effectiveness of the 2016 California Policy to Eliminate Non-Medical Exemptions

on Vaccine Coverage: A Synthetic Analysis <https://github.com/>: Nyathi, Sindiso

Nathan, Lo; 2019 [California Vaccine Policy pre-analysis plan]. Available from: <https://github.com/NathanLo3/Publication-codes/raw/master/California%20Vaccine%20Coverage%20Analysis-%20synth%20control%20pre-analysis%20plan.pdf>.

35.	Lo NC, Nyathi S, Karpel H. California Vaccine Policy Code Repository <https://github.com/SindisoNyathi/California-Vaccine-Policy2019> [Available from: <https://github.com/SindisoNyathi/California-Vaccine-Policy>.

36.	California Vaccine Policy Data [Internet]. 2019. Available from: <https://figshare.com/articles/California_Vaccine_Policy_Data/9775496>.

*Comment #3*

Please revise your title according to PLOS Medicine's style. Your title must be nondeclarative and not a question. It should begin with main concept if possible. "Effect of" should be used only if causality can be inferred, i.e., for an RCT. Please place the study design ("A randomized controlled trial," "A retrospective study," "A modelling study," etc.) in the subtitle (ie, after a colon).

Response: We have updated the title to be in accordance with the PLOS Medicine’s style.

-Article Title:

**Impact of the 2016 California Policy to Eliminate Non-Medical Exemptions on Vaccine Coverage: An Empirical Policy Analysis**

*Comment #4*
Abstract: Please quantify the main results with 95% CIs and p values.

Response: We appreciate the editor’s attention to reporting of our main results; for the county level analysis, we have now added the 95% CI and p-values in the abstract, manuscript results section, and Table 1. For statistical reporting of the synthetic control results, we describe this further in Comment #8 below. While we added p-values, per recent guidance from the American Statistical Association, we would prefer to only include 95% CI to reduce reliance on p-value interpretation alone, although we defer to the editor (ref: https://www.amstat.org/asa/files/pdfs/P-ValueStatement.pdf).

-In Abstract

In the state level synthetic control analysis, MMR coverage in California increased by 3.3% relative to its synthetic control in the post-policy period (top 2 of 43 states, top 5%). Non-medical exemptions decreased by 2.4% (top 2 of 43 states, top 5%), while medical exemptions increased by 0.4% (top 1 of 44 states, top 2%). In the county level analysis, overall vaccination coverage increased by 4.3% **(95% CI 2.9-5.8, p<0.001**), non-medical exemptions decreased by 3.9% (**95% CI 5.4-2.4, p<0.001**), and medical exemptions increased by 2.4% (**95% CI 2.0-2.9, p<0.001).**

-In Table 1

**Table 1: County level analysis of changes in vaccination coverage associated with the 2016 California policy using a difference-in-differences regression**

| **Parameter** | **Overall vaccination coverage (95% CI)^b^** | **p-value** | **Medical exemptions prevalence (95% CI)^b^** | **p-value** | **Non-medical exemptions prevalence (95% CI)^b^** | **p-value** |
| --- | --- | --- | --- | --- | --- | --- |
| **2016 California policy ^a^** | 4.3 (2.9 - 5.8) | <0.001 | 2.4 (2.0 - 2.9) | <0.001 | -3.9 (5.4 - 2.4) | <0.001 |
| **Median income per $10,000 (no)** | 1.0 (0.9 – 2.0) | <0.001 | -0.3 (0.4 - 0.1) | 0.002 | -0.3(0.4 – 0.8) | 0.005 |
| **Mean household size (no)** | -0.1 (-2.3 – 2.1) | 0.9 | 0.5 (-0.3 - 1.3) | 0.3 | 0.2 (-0.6 – 1.0) | 0.7 |
| **Population per 100,000 (no)** | -0.1 (-0.9 – 0.6) | 0.7 | 0.3 (-0.2 – 1.0) | 0.2 | 0.08 (-0.3 - 0.4) | 0.7 |
| **Poverty per 1,000 (%)** | -0.4 (-4.6 – 3.8) | 0.8 | 0.05 (-0.9 - 1.0) | 0.9 | -0.05 (-2.0 – 1.0) | 0.5 |
| **White (%)** | -0.03 (-0.06 – 0.01) | 0.2 | 0.003 (-0.01 - 0.01) | 0.6 | 0.03 (0.01 - 0.04) | 0.001 |
| **Education: Less than high school (%)** | 0.5 (0.2 - 0.7) | <0.001 | -0.09 (0.1 - 0.04) | <0.001 | -0.3 (0.4 - 0.2) | <0.001 |
| **Education: Some college or less (%)** | 0.2 (-0.03 - 0.4) | 0.1 | -0.02 (-0.07 – 0.02) | 0.3 | -0.2 (0.2 - 0.09) | <0.001 |
| **Education: Bachelor's or higher (%)** | 0.07 (-0.2 - 0.3) | 0.7 | -0.01 (-0.06 – 0.05) | 0.8 | -0.1 (0.2 - 0.09) | <0.001 |
| **Uninsured children (%)** | 0.08 (-0.01 - 0.2) | 0.1 | 0.06 (0.04 - 0.08) | <0.001 | 0.09 (0.04 - 0.1) | <0.001 |

^a^Difference-in-differences estimates represent relative change in county level vaccination and exemption prevalence for children entering kindergarten in California before and after the 2016 policy compared with children entering kindergarten before and after the policy in counties from control states

^b^Robust standard errors, clustered by county

*Comment #5*
Abstract: Please address the study implications without overreaching what can be concluded from the data; the phrase "In this study, we observed ..." may be useful. Specifically, please avoid causal language such as “The California policy resulted in…”.

Response: We appreciate the editor’s comment and have changed the wording in the abstract and discussion to avoid causal language.

-In Abstract

“**Conclusions: In this study, we observed** **an increase in vaccination coverage and a reduction in non-medical exemptions at state and county levels following the implementation of the California policy.** The observed increase in medical exemptions was offset by the larger reduction in non-medical exemptions. The largest increases in vaccine coverage were observed in the most “high risk” counties, i.e. those with the lowest pre-policy vaccine coverage. Government policies removing non-medical exemptions can be effective at increasing vaccination coverage.

*Comment #6*
Abstract: Please avoid vague statements such as "these results have major implications for policy/clinical care". Mention only specific implications substantiated by the results. Specifically, the phrase “meaningful increase” in the Conclusion section is vague.

Response: We appreciate the opportunity to refine our language to be more specific to our study results. We have modified the Abstract and manuscript to exclude vague statements and replaced these with more specific, targeted language.

-In Abstract

“**Conclusions: In this study, we observed** **an increase in vaccination coverage and a reduction in non-medical exemptions at state and county levels following the implementation of the California policy.** The observed increase in medical exemptions was offset by the larger reduction in non-medical exemptions. The largest increases in vaccine coverage were observed in the most “high risk” counties, i.e. those with the lowest pre-policy vaccine coverage. Government policies removing non-medical exemptions can be effective at increasing vaccination coverage.

*Comment #7*

At this stage, we ask that you include a short, non-technical Author Summary of your research to make findings accessible to a wide audience that includes both scientists and non-scientists. The Author Summary should immediately follow the Abstract in your revised manuscript. This text is subject to editorial change and should be distinct from the scientific abstract. Please see our author guidelines for more information: [https://journals.plos.org/plosmedicine/s/revising-your-manuscript#loc-author-summary](https://urldefense.proofpoint.com/v2/url?u=https-3A__journals.plos.org_plosmedicine_s_revising-2Dyour-2Dmanuscript-23loc-2Dauthor-2Dsummary&d=DwMF-g&c=j5oPpO0eBH1iio48DtsedeElZfc04rx3ExJHeIIZuCs&r=jNVYd06SCcLbSRqlBGRkHKTrz-mf_GKF6gZEjEktpRA&m=8_dfiUx3mh1kmDZNgk_GFJRWKCMKpwoLvCNwu_GyHIM&s=NjMuKy9Nlyxfj7DlNIe_4U0ClHvgGpNFAnfhoBwwFdA&e=)

Response: We have reviewed the PLOS Medicine guidelines on the Author summary and have added the relevant text after the abstract.

-In Author Summary

**Why was this study done?**

- Vaccine hesitancy, parental reluctance or refusal to vaccinate their children, is a growing challenge in public health, and substantial debate has surrounded the role of state level vaccination policies to address this problem.
- Limited empirical research has evaluated the impact of state vaccination policies on vaccination coverage in children.

**What did the researchers do and find?**

- We evaluated the 2016 California vaccine policy that eliminated non-medical childhood vaccination exemptions to understand how state policies can affect vaccine coverage.
- We applied a synthetic control analysis with state level data and a difference-in-differences analysis with county level data to estimate the relationship between the California vaccine policy and changes in MMR or overall vaccine coverage, non-medical exemptions, and medical exemptions.
- At the state level, the California vaccine policy was associated with a 3.3% increase in MMR vaccination coverage, a 0.4% increase in medical exemptions, and a 2.4% decrease in non-medical exemptions.
- At the county level, the California vaccine policy was associated with a 4.3% increase in overall vaccination, a 2.4% increase in medical exemptions, and a 3.9% decrease in non-medical exemptions.

**What do these findings mean?**

- In this study of the 2016 California policy to eliminate non-medical childhood vaccination exemptions, we find that state policies are an effective tool to increase vaccination coverage.

*Comment #8*
Results: Please provide the CIs and p values for the comparison of actual vs synthetic CA analysis and county-level analysis results for post-policy changes in vaccine coverage and medical/nonmedical exemptions. Please also provide p-values for Table 1.

Response: We have now provided 95% confidence intervals and p-values for the county level difference-in-differences analysis throughout the manuscript and in Table 1.

We appreciate the editor’s attention to the reporting of the results of the state level synthetic control analysis. While conventional regression models provide confidence intervals and p-values based on frequentist assumptions, inference in synthetic control methods is grounded in placebo tests (also known as permutation tests). In placebo tests, we re-evaluate the effect size under the null condition (i.e. in all untreated states). These resulting placebo effect sizes quantify the variation in the outcome under the null hypothesis. By comparing them to the actual (California) effect size we can determine whether or not the observed effect size in the treated unit is meaningful or if it is similar in magnitude to the variation in the outcome in the absence of a treatment. The latter case would imply that our intervention or policy is not, in fact, effective.

In other words, placebo testing evaluates whether the effect size observed in the treated group (i.e. California) is larger than that observed in untreated or control groups (in our case, all non-California states). We pre-specified a “statistically meaningful” relationship as an estimated change in vaccine coverage in the top 5% of all placebo tests. This is described in Rehkopf and Basu (<https://www.ncbi.nlm.nih.gov/pubmed/29613871>) with a succinct discussion of the use of placebo tests in synthetic controls, with Hahn (<https://economics.ucr.edu/repec/ucr/wpaper/201802.pdf>) providing a more detailed and technical treatment.

We have modified the Methods and Discussion sections of the manuscript to clarify this important point as suggested by the editor.

- In Methods:

To assess whether the effect size in the synthetic control analysis was meaningful, we used placebo testing. **While conventional regression models provide confidence intervals and p-values based on frequentist assumptions, inference in synthetic control methods is grounded in placebo tests (also known as permutation tests). In placebo tests, we re-evaluate the effect size under the null condition (i.e. in all untreated states). These resulting placebo effect sizes quantify the variation in the outcome under the null hypothesis. By comparing them to the actual (California) effect size we can determine whether or not the observed effect size in the treated unit is meaningful or if it is similar in magnitude to the variation in the outcome in the absence of a treatment. The latter case would imply that our intervention or policy is not, in fact, effective [31, 32]. To conduct the placebo tests, we individually re-assigned treatment status to each of the other states in the control pool**. We then created a synthetic control for the new state and assessed the resulting effect size for every state in the dataset. We compared effect sizes for each state relative to California for all outcomes (S1 Appendix). We prespecified an effect size as meaningful if California was in the top 5^th^ percentile of states.

-In Discussion

**We used placebo tests in our state level synthetic control analysis to evaluate whether the estimated changes in study outcomes (e.g. vaccine coverage) associated with the California policy were statistically meaningful. Placebo tests in synthetic controls compare the effect sizes from untreated units with the effect size in the treated unit. We prespecified that an effect size for California in the top 5^th^ percentile of states would suggest a meaningful difference, i.e. an effective policy [31]. The observed increase in MMR coverage in California associated with the 2016 policy was much greater than the changes in the majority of the placebo states and met our pre-specified threshold for a significant finding (Fig 3, S3 Fig).** However, we also observed notable changes in a select number of placebo states.

*Comment #9*
Figure S1: Please define the abbreviation “RMSPE“. Also, please show the axis beginning at zero. If this is not possible, please show a break in the axis.

Response: We have removed this abbreviation and provide clarification in the figure legend for Fig S1 as suggested by the editor.

-In S1 Fig

**S1 Fig: Characteristic state covariate selection cutoffs**

In the variable selection procedure we used a training (2011, 2012, 2013) and testing set (2014, 2015) to choose the best variable combination. **We used stepwise variable selection on the training dataset to create a synthetic control and evaluated the fit using the testing set, and the resulting error calculated as the Root Mean Square Predictive Error (RMSPE) value (S1 Appendix).** We iteratively added variables to the model and chose a set of covariates that minimized the RMSPE with as few covariates as possible.

*Comment #10*
Figure S2: Please show the axis beginning at zero. If this is not possible, please show a break in the axis.

Response: We have also modified the axes of the plot to start at 0 as recommended.

-In Fig. S2:

**S2 Fig: Cross validation of synthetic controls using training and testing data for variable selection.**

For each characteristic covariate combination, we evaluated the model using a cross-validation procedure of the pre-policy data. As described in the Appendix methods, we separated the pre-policy data into training and test set; we made model predictions for each combination of covariates on the observed data for California for 2014 and 2015. These predictions were used to calculate a Root Mean Square Predictive Error (RMSPE) value to inform variable selection for the final model and to prevent over-fitting the model.

*Comment #11*
Table S7: Please specify if these values are percentages.

Response: We have edited Table S7 as suggested to specify that the values are percentages.

-In Online Material

**S7 Table: Pre-Policy trends for outcome variables in California and control states**

| **Year** | **Average Overall Vaccination Coverage (California)** | **Average Overall Vaccination Coverage (Control States)** | **Average Medical Exemptions (California)** | **Average Medical Exemptions (Control States)** | **Average Non-Medical Exemptions (California)** | **Average Non-Medical Exemptions (Control States)** |
| --- | --- | --- | --- | --- | --- | --- |
| 2010 | 87.8**%** | 94.4**%** | 0.2**%** | 0.8**%** | 4.2**%** | 1.1**%** |
| 2011 | 87.5**%** | 94.4**%** | 0.2**%** | 0.7**%** | 5.1**%** | 1.2**%** |
| 2012 | 86.6**%** | 94.4**%** | 0.2**%** | 0.8**%** | 6.0**%** | 1.7**%** |
| 2013 | 87.1**%** | 94.3**%** | 0.1**%** | 0.6**%** | 6.2**%** | 2.0**%** |
| 2014 | 88.4**%** | 94.2**%** | 0.2**%** | 0.6**%** | 5.4**%** | 2.0**%** |
| 2015 | 90.3**%** | 94.6**%** | 0.2**%** | 0.6**%** | 5.3**%** | 2.0**%** |

To evaluate similarity between California and control states in pre-policy trends, we calculated average county level vaccination coverage and exemption **percentages** provided by state health departments for the pre-policy period.

*Comment #12*
Tables S9 and S10: Please provide any p-values associated with 95% CIs.

Response: We have updated both Table S9 and S10 to include p-values as suggested.

-In Online Material

**S9 Table: County level sensitivity analysis (leave-one-out tests)**

| **Excluded State** | **Overall Vaccination Coverage** | **p-value** | **Medical Exemptions** | **p-value** | **Non-Medical Exemptions** | **p-value** |
| --- | --- | --- | --- | --- | --- | --- |
| North Dakota | 4.38 (2.93 - 5.83) | <0.001 | 2.44 (1.99-2.89) | <0.001 | -3.92 (5.41-2.43) | <0.001 |
| New York | 4.36 (2.90- 5.81) | <0.001 | 2.44 (1.98-2.90) | <0.001 | -4.00 (5.49-2.51) | <0.001 |
| Minnesota | 4.28 (2.82 - 5.73) | <0.001 | 2.45 (2.00-2.90) | <0.001 | -3.91 (5.40-2.42) | <0.001 |
| New Jersey | 4.34 (2.88 - 5.80) | <0.001 | 2.45 (2.00-2.90) | <0.001 | -3.93 (5.52-2.44) | <0.001 |
| Rhode Island | 4.36 (2.91 - 5.81) | <0.001 | 2.44 (1.99-2.89) | <0.001 | -3.95 (5.44-2.46) | <0.001 |
| Oregon | 4.41 (2.95 - 5.87) | <0.001 | 2.44 (1.99-2.89) | <0.001 | -3.90 (5.38-2.42) | <0.001 |
| Maryland | 4.46 (3.01 - 5.91) | <0.001 | 2.47 (2.02-2.92) | <0.001 | -3.96 (5.45-2.47) | <0.001 |
| Massachusetts | 4.39 (2.94 - 5.82) | <0.001 | 2.44 (1.99-2.89) | <0.001 | -3.96 (5.45-2.47) | <0.001 |
| Texas | 3.90 (2.42 - 5.38) | <0.001 | 2.65 (2.19-3.11) | <0.001 | -3.93 (5.44-2.42) | <0.001 |
| Virginia | 4.23 (2.76 - 5.70) | <0.001 | 2.45 (2.00-2.90) | <0.001 | -3.94 (5.43-2.45) | <0.001 |
| Iowa | 4.27 (2.81 - 5.73) | <0.001 | 2.44 (1.99-2.89) | <0.001 | -3.94 (5.43-2.45) | <0.001 |
| Florida | 4.52 (3.06 - 5.98) | <0.001 | 2.16 (1.71-2.61) | <0.001 | -3.82 (5.29-2.35) | <0.001 |
| Connecticut | 4.34 (2.88 - 5.80) | <0.001 | 2.45 (2.00-2.90) | <0.001 | -3.94 (5.43-2.45) | <0.001 |
| Kansas | 4.38 (2.93 - 5.83) | <0.001 | 2.45 (2.00-2.90) | <0.001 | -3.93 (5.42-2.44) | <0.001 |
| Washington | 4.26 (2.83 - 5.69) | <0.001 | 2.47 (2.02-2.92) | <0.001 | -3.86 (5.35-2.37) | <0.001 |
| Arizona | 4.34 (2.89 - 5.79) | <0.001 | 2.48 (2.03-2.93) | <0.001 | -4.00 (5.49-2.51) | <0.001 |
| Arkansas | __ | __ | 2.44 (1.99-2.89) | <0.001 | -3.95 (5.44-2.46) | <0.001 |

We evaluated the influence of states included in the control pool for the county level difference-in-differences analysis to ensure that no single state had a disproportionate influence on the effect size. We iteratively re-ran the model, excluding a single state from the control pool, and reevaluated the effect size. The resulting range of effect sizes suggests that no single state was driving the effect size.

**S10 Table: County level sensitivity analysis with subset of data reporting overall vaccine coverage**

| **Parameter** | **Overall vaccination coverage (95% CI)b** | **p-value** | **Medical exemptions prevalence (95% CI)b** | **p-value** | **Non-medical exemptions prevalence (95% CI)b** | **p-value** |
| --- | --- | --- | --- | --- | --- | --- |
| **2016 California policy ^a^** | 4.1 (2.6 – 5.6) | <0.001 | 2.7 (2.2 – 3.2) | <0.001 | -3.9 (5.4 - 2.4) | <0.001 |
| **Median income per $10,000 (no)** | 1.2 (0.6 – 1.8) | <0.001 | -0.4 (0.7 - 0.2) | 0.001 | -0.3(0.6 – 0.05) | 0.02 |
| **Mean household size (no)** | -1.9 (-5.0 – 1.2) | 0.2 | 1.5 (0.2 – 2.7) | 0.02 | 0.1 (-1.1 – 1.4) | 0.9 |
| **Population per 100,000 (no)** | 0.08 (-0.9 – 1.1) | 0.9 | 0.4 (-0.2 – 1.1) | 0.2 | -0.01 (-0.5 - 0.5) | 1.0 |
| **Poverty per 1,000 (%)** | 0.7 (-4.2 – 5.7) | 0.8 | -0.3 (-1.6 - 1.1) | 0.7 | -1.6 (-3.5 – 0.4) | 0.1 |
| **White (%)** | -0.02 (-0.08 – 0.03) | 0.4 | 0.02 (-0.003 - 0.05) | 0.1 | 0.03 (0.002 - 0.06) | 0.04 |
| **Education: Less than high school (%)** | 0.4 (0.1 - 0.7) | 0.01 | -0.1 (0.2 - 0.04) | 0.002 | -0.3 (0.5 - 0.2) | <0.001 |
| **Education: Some college or less (%)** | 0.08 (-0.2 - 0.4) | 0.5 | -0.04 (-0.1 – 0.03) | 0.3 | -0.2 (0.3 - 0.06) | 0.005 |
| **Education: Bachelor's or higher (%)** | -0.03 (-0.2 - 0.3) | 0.9 | 0.01 (-0.07 – 0.08) | 0.9 | -0.1 (0.2 - 0.06) | 0.004 |
| **Uninsured children (%)** | -0.1 (-0.2 - 0.02) | 0.1 | 0.1 (0.08 - 0.2) | <0.001 | 0.1 (0.06 - 0.2) | <0.001 |

^a^Difference-in-differences estimates represent relative change in county level vaccination and exemption prevalence for kindergartners in California before and after the 2016 policy

^b^Robust standard errors, clustered by county

Note: We reran the difference-in-differences regression using only states who reported overall vaccination coverage. Five states (North Dakota, Rhode Island, Texas, Maryland, and Minnesota) reported MMR coverage only and were excluded. The results suggest using MMR coverage as a proxy for overall coverage for states who did not report overall coverage did not change the effect sizes for our outcome variables.

*Comment #13*
13. Please ensure that the study is reported according to the relevant guidelines, which can be found here: [http://www.equator-network.org/](https://urldefense.proofpoint.com/v2/url?u=http-3A__www.equator-2Dnetwork.org_&d=DwMF-g&c=j5oPpO0eBH1iio48DtsedeElZfc04rx3ExJHeIIZuCs&r=jNVYd06SCcLbSRqlBGRkHKTrz-mf_GKF6gZEjEktpRA&m=8_dfiUx3mh1kmDZNgk_GFJRWKCMKpwoLvCNwu_GyHIM&s=hykbsiNAB9jP7R1SgRHDyyfHWf-9Zti6wsJ9cCK1RTg&e=)
Please include the completed STROBE, RECORD, etc. checklist as Supporting Information. When completing the checklist, please use section and paragraph numbers, rather than page numbers. Please add the following statement, or similar, to the Methods: "This study is reported as per the Strengthening the Reporting of Observational Studies in Epidemiology (STROBE) guideline (S1 Checklist)."

Response: We have added the reporting statement and reference to the STROBE checklist in the Methods section. We have also included a completed STROBE checklist as S4 Appendix to the Supplementary Materials.

-In Methods

Our study objectives, methods, and planned analyses were pre-specified in a pre-analysis plan (S3 Appendix) [34]. **This study is reported as per the Strengthening the Reporting of Observational Studies in Epidemiology (STROBE) guidelines (S4 Appendix).** All statistical analyses were conducted in R (Version 3.5.1). Public datasets and analytical materials are available online (see Appendix) [35, 36]. This was not human subjects research as the study relied on publicly available, aggregated data and was exempt from Institutional Review Board approval.

**Response to Reviewer #1**

*General Comment:* This paper uses routine data-sets to evaluate the impact of a policy change on vaccination coverage in California. I was asked for a statistical report and I interpret that to include all aspects of the design and conduct of the study.

Response: We thank Reviewer #1 for their helpful comments and attention to the methodology in our manuscript. We appreciate the opportunity to address these comments below.

**Points of detail**

*Comment #1*

Page 6: I wonder whether, for an international audience, we need an explanation of what a kindergarten is in the United States context and how it fits into the overall educational system? For instance are they compulsory? Are they the sole gateway to later education?

Response: We appreciate the reviewer’s suggestion to better explain the study group of kindergarten children and their role in the US education system. We have expanded our Methods section to clarify these points.

-In Methods

**In both the state and county level analyses we used data for children entering kindergarten. Kindergarten is the school grade for children aged 5-6 years in the United States, and commonly the entry level into school. It is the most likely to be affected by the policy change given the age of the children in relation to immunization requirements. Although the California policy also requires younger aged children in preschool or daycare to have completed all the relevant vaccinations, kindergarten is the beginning of formal education and, as such, local and state health departments routinely collect immunization data for children in kindergarten.**

*Comment #2*

Page 6: One analysis uses MMR coverage, the other uses overall coverage. There is probably a good reason for this but we should be told. Did the policy change only affect medical exemptions for MMR? If overall vaccination coverage is important what exactly does it cover over and above MMR?

Response: We appreciate the opportunity to clarify the difference in state and county level primary study outcomes. The difference was based on data availability and pre-specified in our pre-analysis plan. For the county level analysis, since data was individually requested from state health departments, we were able to retrieve overall coverage data for counties in the states included in the analysis. However, state level data reported to the CDC only reported MMR coverage and did not include overall coverage. We assume that MMR coverage is a good proxy for overall vaccine coverage and included this as our pre-specified outcome for the state level analysis.

-In Methods

**The primary outcome was MMR vaccine coverage in the state level analysis, and overall coverage in the county level analysis. This difference in primary outcomes between analyses was based on data availability, as states do not report overall coverage to the CDC. We assumed state level MMR coverage was a good proxy for overall coverage, and pre-specified this in our pre-analysis plan (S3 Appendix). Furthermore, because the definitions of medical and non-medical exemptions include children with exemptions for any vaccine and not just the MMR vaccine, for any given state the three outcomes did not always sum to one.**

*Comment #3*

Page 7 If placebo testing is the accepted phrase in the synthetic control world then fine but otherwise it seems a misnomer to me.

Response: We appreciate the reviewer’s attention to “placebo testing”, which is a phrase specific to the synthetic control method. We discuss the use of placebo tests in synthetic controls in Editor’s comments, Comment #8. To address the reviewer’s concern, we have clarified the use of this term throughout the manuscript.

-In Methods

To assess whether the effect size in the synthetic control analysis was meaningful, we used placebo testing**. While conventional regression models provide confidence intervals and p-values based on frequentist assumptions, inference in synthetic control methods is grounded in placebo tests (also known as permutation tests). In placebo tests, we re-evaluate the effect size under the null condition (i.e. in all untreated states). These resulting placebo effect sizes quantify the variation in the outcome under the null hypothesis. By comparing them to the actual (California) effect size we can determine whether or not the observed effect size in the treated unit is meaningful or if it is similar in magnitude to the variation in the outcome in the absence of a treatment. The latter case would imply that our intervention or policy is not, in fact, effective [31, 32]. To conduct the placebo tests, we individually re-assigned treatment status to each of the other states in the control pool. We then created a synthetic control for the new state and assessed the resulting effect size for every state in the dataset. We compared effect sizes for each state relative to California for all outcomes (S1 Appendix).** We prespecified an effect size as meaningful if California was in the top 5^th^ percentile of states.

-In Discussion

**We used placebo tests in our state level synthetic control analysis to evaluate whether the estimated changes in study outcomes (e.g. vaccine coverage) associated with the California policy were statistically meaningful. Placebo tests in synthetic controls compare the effect sizes from untreated units with the effect size in the treated unit. We prespecified that an effect size for California in the top 5^th^ percentile of states would suggest a meaningful difference, i.e. an effective policy [31]. The observed increase in MMR coverage in California associated with the 2016 policy was much greater than the changes in the majority of the placebo states and met our pre-specified threshold for a significant finding (Fig 3, S3 Fig). However, we also observed notable changes in a select number of placebo states.** North Dakota had an increase in coverage of 3.6% relative to its synthetic control during the year of the policy—0.3% larger than the increase in California attributable to the policy.

*Comment #4*

Page 9: It might be better to choose a repository which issues a DOI or one belonging to a university. I understand GitHub has quite a steep learning curve.

Response: We have moved the study data to a Figshare repository, and updated the citations to include the DOI as per PLOS Medicine’s Data sharing policies.

Reference:

34.	Nyathi S, Nathan L. Effectiveness of the 2016 California Policy to Eliminate Non-Medical Exemptions

on Vaccine Coverage: A Synthetic Analysis <https://github.com/>: Nyathi, Sindiso

Nathan, Lo; 2019 [California Vaccine Policy pre-analysis plan]. Available from: <https://github.com/NathanLo3/Publication-codes/raw/master/California%20Vaccine%20Coverage%20Analysis-%20synth%20control%20pre-analysis%20plan.pdf>.

35.	Lo NC, Nyathi S, Karpel H. California Vaccine Policy Code Repository <https://github.com/SindisoNyathi/California-Vaccine-Policy2019> [Available from: <https://github.com/SindisoNyathi/California-Vaccine-Policy>.

36.	California Vaccine Policy Data [Internet]. 2019. Available from: <https://figshare.com/articles/California_Vaccine_Policy_Data/9775496>.

*Comment #5*

Page 16: Figure 4 Since the x–axis variable is presumably negatively correlated with part of the composite y–axis variable is there some artefact here as well as any possible true effect?

Response: We appreciate the opportunity to clarify Figure 4. We have changed Figure 4 to address the point that in counties with higher non-medical exemption prevalence in 2015, the increase in overall vaccination coverage from 2015 to 2017 could be due to regression to the mean and is bounded based on this relationship. We now graph the changes in overall coverage, medical, and non-medical exemptions as a function of each outcome’s pre-policy (2015) prevalence to provide broader data for interpretation.

In Results:

**Fig 4: Post-policy changes in county level vaccination coverage and exemptions as a function of pre-policy prevalence.**

This figure plots the change in overall vaccination coverage and exemptions before and after the policy for each county in California as it related to their pre-policy (2015) prevalence. In counties with greater pre-policy prevalence of non-medical exemptions, there were larger decreases in non-medical exemptions following the policy. Counties with lower pre-policy overall vaccination coverage had greater changes in overall coverage following the policy’s implementation. Please note the axis magnitudes are different for each plot based on baseline magnitude of outcome.

-In Results

There was substantial variation in vaccination coverage and exemptions across counties before and after the policy implementation. **Counties with a higher proportion of pre-policy non-medical exemptions in 2015 (i.e. “hot spots” most at risk of outbreaks) had larger decreases in non-medical exemptions following the policy’s implementation (Fig 4). Likewise, counties with lower pre-policy overall coverage had the largest increases in overall coverage following the policy’s implementation. Although our model found an absolute increase in medical exemptions in counties of 2.4%, we did not see a strong trend in relation to pre-policy medical exemption percentage (Fig 4).**

*Comment #6*

References, Some of these could use checking. Reference 31 looks rather incomplete for example.

Response: We appreciate the attention to the references, and have now checked and corrected all the references, including those related to Acts and Bills.

In References:

13.	California Legislative Information. SB-277 Public health: vaccinations. (2015-2016) <https://leginfo.legislature.ca.gov/faces/home.xhtml>: California Legislative Information,; 2019 [Available from: <https://leginfo.legislature.ca.gov/faces/billNavClient.xhtml?bill_id=201520160SB277>.

35.	Lo NC, Nyathi S, Karpel H. California Vaccine Policy Code Repository <https://github.com/SindisoNyathi/California-Vaccine-Policy2019> [Available from: <https://github.com/SindisoNyathi/California-Vaccine-Policy>.

38.	Vermont General Assembly. H.98 (Act 37): An act relating to reportable disease registries and data. <https://legislature.vermont.gov/>: Vermont General Assembly,; 2015 [Available from: <https://legislature.vermont.gov/bill/status/2016/H.98>.

*Comment #7*

I see from that invaluable resource the internet that kindergartner is used in the US for children attending a kindergarten. In some places the authors use the phrase children attending kindergarten which might be preferable to help those German speakers who use Kinderg¨artner(in) to mean an employee at a Kindergarten

Response: We enjoy the reviewer’s thoughtful attention to linguistically appropriate use of kindergartener. We now rephrase this usage to say “children entering kindergarten” as suggested by the reviewer. We have provided a sampling of these changes below.

-In Methods:

The primary outcome for the state level analysis was pre-specified as measles, mumps, and rubella (MMR) vaccine coverage, defined as the proportion of **children entering kindergarten** who have received the 2-dose MMR vaccine by the start of the school year. The secondary outcomes were the proportion of **children entering kindergarten** with medical and non-medical exemptions at the start of the school year.

-In Table 1 caption

^a^Difference-in-differences estimates represent relative change in county level vaccination and exemption prevalence for **children entering kindergarten** in California before and after the 2016 policy compared with **children entering kindergarten** before and after the policy in counties from control states

^b^Robust standard errors, clustered by county

**Points of more substance**

*Comment #8*

The synthetic control model I am not familiar with synthetic control models, an ignorance which I suspect I might share with many readers of the journal. I think a few more details might help. The description in the appendix leaves open the question of where the weights come from. Are these coefficients from the regressions? That seems very unlikely so where do they come from?

Response: We thank the reviewer for their attention to the construction of the synthetic control for California through a weighted average of other states. The synthetic control method uses an optimization algorithm that estimates weight (i.e. coefficients) for all control states to minimize the difference between the treated state (California) and the synthetic control (weighted average of other states) in the pre-treatment time period (2011 – 2015). Of note, the weight for many states is often zero and the states are effectively removed from construction of the synthetic control. The resulting synthetic California (i.e. the resulting combination of weights) is one that minimizes the error between itself and actual California in the pre-treatment period. To address the reviewer’s important point, we have added text in the Methods and Appendix to explain this as shown below. We have further reviewed the entire manuscript and clarified these points where relevant.

- In Methods

We used a synthetic control study design to estimate the relationship between the California policy and vaccination coverage and exemptions at the state level. **The synthetic control method is a statistical tool designed for comparative case studies, such as policy evaluations, where only a single treated unit is available (e.g. state level policy)** [28-30]. The approach constructs a hypothetical control state (i.e. a “synthetic control California”) that matches the treated state (i.e. actual California) on the pre-policy outcome**. The synthetic California is constructed with a weighted combination of control states. The synthetic control optimization algorithm estimates a weight for all non-experimental states that minimizes the difference between the actual California and the “synthetic” California in the pre-treatment period. Most states receive a zero weight, and only states with non-zero weights provide information for the construction of the synthetic control.** The resulting synthetic control California provides a counterfactual estimation of the study outcome during the post-policy period in the treated state in the absence of the treatment, i.e. **it projects the outcome in the absence of the policy.**

- In Appendix:

*Synthetic control methodology*

**The synthetic control approach creates a synthetic control California that describes the change in the outcome in the absence of the treatment. The synthetic California is constructed from a weighted combination of all potential control states. The synthetic control optimization algorithm chooses a set of weights that minimize the difference between the actual California and the “synthetic” California in the pre-treatment period. The resulting synthetic control California provides a counterfactual estimation of the study outcome during the post-policy period in the treated state in the absence of the treatment.**

We defined three outcomes for the state level analysis: MMR coverage, prevalence of non-medical exemptions, and prevalence of medical exemptions. In order to be included in the pool of potential control states for a given outcome, a state needed to have complete data records for all study years for that outcome. If a state was missing any data for any year for an outcome it was excluded from the control pool for that outcome. The states excluded from the control pools for each outcome are listed in S2 Table. **Since the synthetic control method minimizes the difference in the outcome between the synthetic California and the actual California in the pre-treatment period, we created three synthetic “California” states, one for each outcome as per common practice.**

Comment #9:

If one wanted to be very unkind one might state that the authors have not used a synthetic California but three very different synthetic Californias, one for each outcome. Given that the outcomes are closely related since they sum to unity this seems strange. Would the authors like to explain this further? The authors have undertaken extensive regression analyses to arrive at their models. This means the coefficients will be very data-driven.

We thank the reviewer for their comment on the construction of three separate synthetic control “Californias” for each outcome (one primary, two secondary), and appreciate the opportunity to clarify this important point. As the reviewer points out, the construction of weights is data driven. The goal of the estimation algorithm is to minimize the difference in the outcome between the resulting synthetic control and the experimental state (e.g. MMR coverage in California). As such, all variable selection, cross validation and estimation of weights for the synthetic controls were conducted for the main outcome, which is MMR coverage *and* repeated for our two secondary outcomes of medical and non-medical exemptions given they are different data and trajectories. This process was pre-specified in our pre-analysis plan. Ultimately, we find that the synthetic controls are similar across outcomes, but the synthetic control is re-constructed for each outcome as per conventional practice. To address the reviewer’s point, we have clarified this in the methods and appendix sections.

-In Methods:

The key effect size for each outcome was the difference in pre- to post-policy change between California (i.e. the treated state) and the synthetic control California (i.e. the hypothetical untreated state). **We constructed a unique synthetic control for each of the three outcomes, as is convention given the data driven nature of the process. This resulted in three synthetic control “California” states; one corresponding to each outcome.**

-In Appendix:

We defined three outcomes for the state level analysis: MMR coverage, prevalence of non-medical exemptions, and prevalence of medical exemptions. In order to be included in the pool of potential control states for a given outcome, a state needed to have complete data records for all study years for that outcome. If a state was missing any data for any year for an outcome it was excluded from the control pool for that outcome. The states excluded from the control pools for each outcome are listed in S2 Table. **Since the synthetic control method minimizes the difference in the outcome between the synthetic California and the actual California in the pre-treatment period, we created three synthetic “California” states, one for each outcome as per common practice.**

Comment #10:

This may or may not matter since the goal is prediction not explanation but I do question how stable the weights are. Would it make much difference if equal weights were used especially for the MMR coverage data? There is an interesting discussion of the use of equal coefficients in Wainer (1976).

Michael Dewey

References

H Wainer. Estimating coefficients in linear models: it don’t make no nevermind. Psychological Bulletin, 83:213–217, 1976.

Response: We appreciate the reviewer’s attention to the question of the stability of the state weights. This is an important aspect of the synthetic control. In general, the resulting synthetic control is not necessarily unique. Perturbations in the control states result in different weights comprising the synthetic control. In theory this could result in different effect sizes. This very concern is addressed by our sensitivity analysis of states included in the control pool. As explained in the methods and in the S2 Appendix, for this sensitivity analysis we re-ran the synthetic control model, iteratively excluding a single state from the donor pool, and re-evaluated the effect size. We found that changes in the control states and therefore the weights did not result in large changes in the resulting effect sizes [3.18% – 4.04% range, Table S5]. This result suggests that while the weights may be variable and the synthetic control may not be unique, the resulting effect sizes fall within a narrow range and the study findings remain robust.

-In Table S5.

**S5 Table: Control state sensitivity analysis with leave-one-out tests.**

| **Excluded State** | **MMR Coverage** | **Non-medical Exemptions** | **Medical Exemptions** |
| --- | --- | --- | --- |
| Alabama | 3.41 | -2.36 | 0.40 |
| Alaska | 3.34 | -2.36 | 0.40 |
| Arizona | 3.28 | -2.36 | 0.41 |
| Arkansas | 3.74 | -2.36 | 0.40 |
| . . . |  |  |  |
| Virginia | 3.23 | -2.36 | 0.40 |
| Washington | 3.26 | -2.37 | 0.40 |
| West Virginia | 3.23 | -2.36 | 0.40 |
| Wisconsin | 3.25 | -2.37 | 0.40 |
| **Reported [Range]** | **3.34**  **[3.18 – 4.04]** | **-2.36**  **[2.77 – 1.92]** | **0.39**  **[0.39 – 0.41]** |

**We evaluated the influence of states included in the control pool to ensure that no single state had a disproportionate influence on the effect size and that slight perturbations of state weights did not significantly change the effect size.** We iteratively re-ran the model, excluding a single state from the control pool, and re-evaluated the effect size. **The resulting range of effect sizes suggests that even with slightly different control pools, and therefore slightly different weights, the effect size remains stable. In addition, no single state was driving the effect size.**

*Comment #11*

County model The differences in differences analysis relies on the parallel trend assumption. This is untestable of course but is there any evidence that the tends were parallel before the intervention?

Response: We appreciate the reviewer’s attention to the assumption on parallel trends to ensure the difference-in-differences analysis is valid. In addition to the S7 Table in Appendix 2 that provides the average trends in outcome variables before the policy went into effect, we have provided plotted data to demonstrate this condition is met in Figure S5 in Appendix 2. We have also added this discussion in the Methods and Discussion/Limitations to highlight this point for the reader.

-In Methods:

We used a difference-in-differences study design to evaluate the association between the California policy and vaccination coverage and exemptions at the county level. The difference-in-differences design estimates the relative change in vaccination coverage and exemptions over time associated with the California policy as the difference between the treated group of counties (California) before and after the policy implementation, and the control counties (counties not in California) before and after the policy implementation. **To assess the parallel trends assumption relevant to the difference-in-differences method, we plotted the data for California and the control counties before the policy’s implementation (S2 Appendix).** We used an ordinary least squares regression model for all outcomes and calculated robust standard errors clustered by county. We included an adjusted analysis with county level characteristics (S2 Appendix).

-In Results:

**In the county level analysis, we found that the trends for outcome variables were relatively similar between California counties and counties in control states in the pre-policy period (S5 Fig).** Overall vaccination was generally similar in the pre-policy period, although California counties had slightly lower vaccination levels and greater variation than control counties. Pre-policy variation in non-medical exemptions for California was greater than for control states (S7 Table).

-In Discussion:

The synthetic control is ideally constructed without any acute changes during the pre-intervention period, although California passed two policies aiming to increase vaccine coverage. In 2014, California passed AB2109 which required proof of having consulted a healthcare provider before receiving an exemption. In 2015, California introduced a second initiative meant to educate administrators on the conditional admissions requirements. The 2015 conditional entrants initiative, while effective, was limited in scope, compared to the statewide policy, and would only affect MMR coverage or overall exemptions, and not non-medical exemptions. In addition, these policies were unlikely to meaningfully affect our estimate since the calibration process of the synthetic control minimized these perturbations [39]. **We plotted pre-policy trends in vaccination coverage and exemptions to assess the parallel trends assumption in the difference-in-differences analysis. Although the trends were similar between California and control counties before the intervention, there was greater variability in California over the years, especially for non-medical exemptions.** Finally, our analyses included only two years of post-policy data, so we are unable to observe the persistence of increased coverage over time.

-In Appendix:

**S5 Fig: Pre-Policy Trends in Outcome Variables in California and Control Counties**

To assess the parallel trends assumption for the county level difference-in-differences analysis, we plotted the average county level vaccination coverage and exemption percentages for California counties and control counties from the 2010-2011 school year through the 2015-2016 school year. The California policy went into effect before the start of the 2016-2017 school year.

*Comment #12*

In the Appendix the authors state ‘As with the state level analysis, our results suggest that no single state disproportionately drives our effect size.’ The bottom two panels in Figure 2 suggest to me that the first six words might be removed from the sentence. I agree it is not just one state driving things there but it is only a handful.

Response: We have revised the appendix as suggested by the reviewer. In response to the distribution of weights of the states used in the synthetic control, please refer to point 1 in Comment #10 above.

-In S1 Appendix:

In the leave-one-out tests, we tested whether any single state was disproportionately driving the effect size. We reran the synthetic control model iteratively excluding a single state from the control pool. We then re-estimated the effect sizes from the resulting models and evaluated the outcome. Results are shown in S5 Table of the supplementary materials. Each synthetic control California is comprised of a modest number of states (3 – 5). Our results suggest that for the synthetic control analysis, no single state disproportionately drives our effect size.

**S5 Table: Control state sensitivity analysis with leave-one-out tests**

| **Excluded State** | **MMR Coverage** | **Non-medical Exemptions** | **Medical Exemptions** |
| --- | --- | --- | --- |
| Alabama | 3.41 | -2.36 | 0.40 |
| Alaska | 3.34 | -2.36 | 0.40 |
| Arizona | 3.28 | -2.36 | 0.41 |
| Arkansas | 3.74 | -2.36 | 0.40 |
| . . . |  |  |  |
| Virginia | 3.23 | -2.36 | 0.40 |
| Washington | 3.26 | -2.37 | 0.40 |
| West Virginia | 3.23 | -2.36 | 0.40 |
| Wisconsin | 3.25 | -2.37 | 0.40 |
| **Reported [Range]** | **3.34**  **[3.18 – 4.04]** | **-2.36**  **[2.77 – 1.92]** | **0.39**  **[0.39 – 0.41]** |

We evaluated the influence of states included in the control pool to ensure that no single state had a disproportionate influence on the effect size **and that slight perturbations of state weights did not significantly change the effect size**. We iteratively re-ran the model, excluding a single state from the control pool, and re-evaluated the effect size. The resulting range of effect sizes suggests that **even with slightly different control pools, and therefore slightly different weights, the effect size remains stable. In addition**, no single state was driving the effect size.

- In S2 Appendix

We performed sensitivity analyses to determine the robustness of our findings. In the leave-one-out test, we reran the model excluding a single state from the control pool. The effect sizes from the resulting models are shown in S9 Table. **Our results suggest that no single state disproportionately drives our effect size.** As such, our effect size is robust to states included in the analysis. Additionally, given the availability of data from control states, we performed a sub-analysis that excluded states which reported MMR coverage and not overall vaccination coverage. The effect sizes from the resulting model are shown in S10 Table.

*Comment #13*

Summary Points for clarification and more explanation of the modelling. The fact that the two different methods give broadly similar results increases credibility.

Response: We appreciate the reviewer’s summary. Indeed, our goal was to use two independent datasets and different statistical methods to test the same hypothesis to ensure robustness of our findings. We have highlighted this important aspect of our study in the Discussion section.

-In Discussion

In this empirical policy analysis, we evaluated California’s 2016 policy removing non-medical vaccine exemptions and found robust evidence that the policy was associated with an increase in vaccination coverage in children entering kindergarten. At the state level, the policy was associated with a 3.3% increase in MMR coverage and 2.4% reduction in non-medical exemptions. The policy was also associated with a modest 0.4% increase in medical exemptions. At the county level, often the counties with high baseline exemption prevalence and most “at risk” of an outbreak had the largest increases in vaccine coverage from the policy. Despite concerns around the observed increase in medical exemptions, our study finds that the policy was associated with an overall increase in vaccine coverage. **The use of two independent state and county level analyses with consistent results provides robust evidence to support the adoption of similar state policies eliminating non-medical exemptions to address the growing public health challenge of vaccine hesitancy.**

-In Conclusion

As vaccine hesitancy becomes a larger public health challenge in the United States and globally, with vaccine-preventable disease outbreaks growing, the debate concerning state policies to remove non-medical exemptions is ongoing. While such policies do not address the larger problem of vaccine hesitancy including a lack of confidence in vaccination, they may effectively increase vaccination coverage. Our study finds that following the California policy eliminating non-medical exemptions, vaccine coverage increased and non-medical exemptions decreased in California. **Our conclusions are strengthened by the use of two independent analyses which both found similar results.** This study supports state level governmental policies to remove non-medical exemptions to increase vaccine coverage across the United States.

**Response to Reviewer #2**

*General comment:*

General comment: This is a well-conducted and well-reported study on the effect of the 2016 California law removing non-medical exemptions in that state. 
There are a few issues that need to be addressed to improve this manuscript.

Response: We thank Reviewer #2 for their kind and helpful comments.

*Comment #1*
In the abstract and in the introduction, you refer to vaccine hesitancy as the delay or refusal of vaccination, but hesitancy can also mean underlying lack of confidence in vaccination, even if vaccines are received.  This may be a bit pedantic, but hesitance does not always equate to refusal or delay.

Response: We appreciate this thoughtful comment from Reviewer #2 on the definition of vaccine hesitancy. We based our original definition from the WHO definition of vaccine hesitancy (<https://www.who.int/immunization/sage/meetings/2014/october/SAGE_working_group_revised_report_vaccine_hesitancy.pdf?ua=1>), and agree with the reviewer that lack of confidence is an important source of lack of vaccination. To address this concern, we have expanded our definition to include lack of confidence in vaccination as suggested.

-In Introduction:

**Vaccine hesitancy, defined as the reluctance or refusal to vaccinate despite the availability of vaccinations, is a growing public health challenge** [1-3]. Declining vaccination rates driven by vaccine hesitancy **and lack of confidence in vaccines** have led to recent outbreaks of vaccine-preventable diseases and threaten the public health gains made against these diseases in past decades [4-6]. The factors driving vaccine hesitancy are complex, but include misconceptions and misinformation about vaccine safety, low perceived risk of infectious diseases, and lack of trust in healthcare providers [7, 8].

-In Discussion:

As vaccine hesitancy becomes a larger public health challenge in the United States and globally, with vaccine-preventable disease outbreaks growing, the debate concerning state policies to remove non-medical exemptions is ongoing. **While such policies do not address the larger problem of vaccine hesitancy including a lack of confidence in vaccination, they may effectively increase vaccination coverage.** Our study finds that following the California policy eliminating non-medical exemptions, vaccine coverage increased and non-medical exemptions decreased in California.

*Comment #2*
Throughout the manuscript, it should be more clear whether the % changes that are presented are relative or absolute changes. It appears they are absolute changes, but as written, it is a bit confusing.

Response: We agree with the reviewer on the importance of clarifying our results. The majority of study results are presented as absolute changes. We have gone through the manuscript to revise accordingly to make this clearer, and include a representative set of changes made:

-In Methods

**All results are presented as absolute percentage changes in MMR coverage, and medical and non-medical exemptions.**

**All results are presented as absolute percentage changes in overall vaccination coverage, and medical and nonmedical exemptions.**

-In Results:

There was substantial variation in vaccination coverage and exemptions across counties before and after the policy implementation. **Counties with a higher proportion of pre-policy non-medical exemptions in 2015 (i.e. “hot spots” most at risk of outbreaks) had larger decreases in non-medical exemptions following the policy’s implementation (Fig 4). Likewise, counties with lower pre-policy overall coverage had the largest increases in overall coverage following the policy’s implementation. Although our model found an absolute increase in medical exemptions in counties of 2.4%, we did not see a strong trend in relation to pre-policy medical exemption percentage (Fig 4). The absolute change in vaccination coverage between 2015 and 2017 across counties in California ranged from -6% to 26% with 12/57 (21.1%) of counties decreasing coverage, 28/57 (49.1%) experiencing an increase in coverage between 0.1-4 percentage points, and 17/57 (29.8%) experiencing an increase in coverage >4 percentage points (S8 Table). For non-medical exemptions, 10/57 (17.5%) of counties in California either had no change or an increase and 47/57 (82.5%) of counties had a decrease in non-medical exemptions ranging from -0.1% to -21.5% between 2015 and 2017. All counties had an absolute increase in medical exemptions, ranging from 0.5% to 9.3%. Plumas county, population 18,742, experienced the 9.3% rise in medical exemptions from 1.0% in 2015 to 10.3% in 2017 (S8 Table).**

*Comment #3*
A little more methodologic explanation of the synthetic control analysis would be helpful, especially for policy makers who may read this who do not have more advanced statistical training.

Response: We agree with the reviewer on the importance to better explain the synthetic control method, especially for key audience members such as policymakers. We have clarified language throughout the manuscript as below. We have also added additional details as discussed in other comments (e.g. Reviewer 1, Comment #8; Editor, Comment #8).

In Methods

We used a synthetic control study design to estimate the relationship between the California policy and vaccination coverage and exemptions at the state level. **The synthetic control method is a statistical tool designed for comparative case studies, such as policy evaluations, where only a single treated unit is available (e.g. state level policy)** [28-30]. The approach constructs a hypothetical control state (i.e. a “synthetic control California”) that matches the treated state (i.e. actual California) on the pre-policy outcome**. The synthetic California is constructed with a weighted combination of control states. The synthetic control optimization algorithm estimates a weight for all non-experimental states that minimizes the difference between the actual California and the “synthetic” California in the pre-treatment period. Most states receive a zero weight, and only states with non-zero weights provide information for the construction of the synthetic control.** The resulting synthetic control California provides a counterfactual estimation of the study outcome during the post-policy period in the treated state in the absence of the treatment, i.e. **it projects the outcome in the absence of the policy.**

- In Appendix:

*Synthetic control methodology*

**The synthetic control approach creates a synthetic control California that describes the change in the outcome in the absence of the treatment. The synthetic California is constructed from a weighted combination of all potential control states. The synthetic control optimization algorithm chooses a set of weights that minimize the difference between the actual California and the “synthetic” California in the pre-treatment period. The resulting synthetic control California provides a counterfactual estimation of the study outcome during the post-policy period in the treated state in the absence of the treatment.**

*Comment #4*
There are some reported results where % change is given without the context of baseline rates. While this is presented well in Figure 4 for the county-level analysis, it would be helpful to ensure this has the best context throughout.

Response: We thank the reviewer for this suggestion to provide baseline values for outcomes when presenting key outcomes. We have modified the key Results and Discussion section to follow this suggestion.

In Results:

In this state level analysis, we estimated that the 2016 California policy was associated with a **3.3% increase in MMR coverage, from 94.5% in 2015 and 92.6% in 2014,** relative to its synthetic control in the post-policy period (top 2 of 43 states, top 5%). The largest improvement in MMR coverage occurred in North Dakota (3.6%). **The 2016 California policy was associated with a 2.4% decrease in non-medical exemptions in the post-policy period (top 2 of 43 states, top 5%), from 2.4% in 2015 and 2.5% in 2014, while medical exemptions increased by 0.4% (top 1 of 44 states, top 2%), from 0.2% in 2015 and 2014 (Fig 3).**

In Results:

Although our model found an absolute increase in medical exemptions in counties of 2.4%, we did not see a strong trend in relation to pre-policy medical exemption percentage (Fig 4). The absolute change in vaccination coverage between 2015 and 2017 across counties in California ranged from -6% to 26% with 12/57 (21.1%) of counties decreasing coverage, 28/57 (49.1%) experiencing an increase in coverage between 0.1-4 percentage points, and 17/57 (29.8%) experiencing an increase in coverage >4 percentage points (S8 Table). For non-medical exemptions, 10/57 (17.5%) of counties in California either had no change or an increase and 47/57 (82.5%) of counties had a decrease in non-medical exemptions ranging from -0.1% to -21.5% between 2015 and 2017. **All counties had an absolute increase in medical exemptions, ranging from 0.5% to 9.3%. Plumas county, population 18,742, experienced the 9.3% rise in medical exemptions from 1.0% in 2015 to 10.3% in 2017 (S8 Table).**

*Comment #5*
In the discussion, third paragraph, it would be helpful to mention that ND and VT are relatively small states, compared to CA, and relatively small changes in absolute numbers of children vaccinated or exempted could make a larger impact than in CA. Additionally, some context as to the overall absolute change, in N, of the children with vaccination or without exemption, would be helpful.

Response: We thank the reviewer for their attention to the high increases in coverage in North Dakota and Vermont. We have included the reviewer’s helpful points in the Discussion as shown below.

-In Discussion.

This is likely due to Vermont’s 2015 policy which removed philosophical exemptions [38]. **Notably, the number of children enrolled in kindergarten in North Dakota and Vermont in 2016 was approximately 10,000 and 6,500 respectively, while the kindergarten enrollment for California in the same year was approximately 521,000 [27]. So despite the slightly larger percentage changes in smaller states like North Dakota and Vermont, the absolute number of children affected is much smaller than in California.** Non-medical exemptions in California showed a slight decrease in 2014. This may have been a result of another California policy passed in 2014 that required parents seeking exemptions to provide signed documentation from a health care provider [39].

**Response to Reviewer #4**

*General Comment:* This analysis evaluates the effectiveness of SB277 in California. There have been two recent publications tackling the exact same question (below). This paper does use different set of methods than those.

Pingali SC, Delamater PL, Buttenheim AM, Salmon DA, Klein NP, Omer SB. Associations of Statewide Legislative and Administrative Interventions With Vaccination Status Among Kindergartners in California. JAMA. 2019;322:49-56.

Delamater PL, Pingali SC, Buttenheim AM, Salmon DA, Klein NP, Omer SB. Elimination of Nonmedical Immunization Exemptions in California and School-Entry Vaccine Status. Pediatrics. 2019;:e20183301.

Response: We appreciate Reviewer #4’s reference to recently published articles that address the California vaccine policy. We now include these references and have added substantial discussion about these articles in the introduction and discussion. As the reviewer mentions, our study applies a different dataset and methodology, including a controlled analysis with additional insights, especially on county level variation. These include robust evidence that despite an increase in medical exemptions, overall vaccination still increased following the policy’s implementation at both the state and county level. Further, we explore county level variation in effect size in our paper.

-In Introduction

**While previous observational studies have suggested that coverage increased in California after the policy’s implementation, the policy’s effectiveness remains unclear given natural variation in vaccination rates and lack of controlled policy evaluation** [15-18]. Furthermore, the rate of medical exemptions in California increased after the policy, causing concerns that children who had received non-medical exemptions were instead receiving medical exemptions, thus limiting the policy’s ability to increase overall vaccine coverage [7, 19].

-In Discussion:

**Two recent studies provided additional descriptive analyses of the impact of the California vaccine policies on vaccination coverage [17, 18]. In one study, the authors used county level data for California to report the changes in the percentage of children entering kindergarten with up-to-date vaccination status, as well as geographic clustering of unvaccinated children associated with three California vaccination policies. In a subsequent study, the authors extended this work to focus specifically on SB277, the 2016 California vaccine policy. Both studies found an increased percentage of children entering kindergarten with all vaccinations completed post implementation of the California policy, though attribution to SB227 is compromised by the limited availability of comparison units, leaving open the possibility that observed changes may be over- or under-estimated, depending on the secular trends. Our study further extended this work by providing a controlled, quasi-experimental methodological approach to estimate the impact of the 2016 California vaccine policy. Taken together, these studies contribute robust evidence to support the conclusion that vaccination coverage increased after the California policy’s implementation.**

-In Discussion

As multiple states continue to experience outbreaks of vaccine preventable diseases, interventions like the California policy that increase vaccine coverage levels above the required thresholds will continue to be vital. **A recent analysis of the California policy suggested that legislation may need to consider local differences, as areas of Northern California continued to demonstrate high rates of students without up-to-date vaccination status despite the state’s intervention [17]. Our county level analysis found a relationship between income and education and vaccine coverage, further demonstrating the complexity of this relationship. Additional research into other correlates of vaccine hesitancy in particular regions may be necessary to obtain ideal coverage in areas that remain vaccine hesitant.** Finally, settings that remain at low vaccine coverage despite removal of non-medical exemptions may require additional interventions that include local community involvement and educational programs [40-42].

-In References:

**15.	Delamater PL, Leslie TF, Yang YT. Change in Medical Exemptions From Immunization in California After Elimination of Personal Belief Exemptions. JAMA. 2017;318(9):863-4.**

**17.	Pingali SC, Delamater PL, Buttenheim AM, Salmon DA, Klein NP, Omer SB. Associations of Statewide Legislative and Administrative Interventions With Vaccination Status Among Kindergartners in California. JAMA. 2019;322(1):49-56.**

Specific concerns/issues/comments

*Comment #1*
The synthetic control approach is very interesting, but I do have some reservations about its validity in this particular scenario. There are numerous factors that can and do influence state-level UTD rates, but specifically policy changes are shocks that may interrupt and/or change prior temporal trends. In California, prior to SB277, the state enacted AB2109 two years prior to SB277, which interrupted temporal trends in the year prior (note the small decreases in NMEs in 2014 and 2015 in Fig 1. It seems that constructing a synthetic control without accounting for the different policy regimes in the potential control data over the time period (i.e., other states) may lead to an inaccurate estimate of what we should have expected in CA.

Response: We appreciate this thoughtful point from reviewer #4 on the synthetic control calibration, and how changes during the pre-policy period may affect the results. We agree with the reviewer that the synthetic control is ideally constructed without acute changes during the pre-policy period. Notably, the calibration procedure of the synthetic control construction will minimize differences between California and the constructed synthetic control, and thus will absorb small perturbations in the trajectory of the outcome. We estimate this perturbation to be at most estimated a 0.8% change, which corresponds to the estimated increase in coverage as a result of the 2014 policy (Buttenheim et al., 2018. <https://www.ncbi.nlm.nih.gov/pubmed/29778514>). To address the reviewer’s valid concern, we have added the reviewer’s point to the Methods and Limitations subsection of the discussion.

-In Discussion (Limitations)

**The synthetic control is ideally constructed without any acute changes during the pre-intervention period, although California passed two policies aiming to increase vaccine coverage. In 2014, California passed AB2109 which required proof of having consulted a healthcare provider before receiving an exemption. In 2015, California introduced a second initiative meant to educate administrators on the conditional admissions requirements. The 2015 conditional entrants initiative, while effective, was limited in scope compared to the statewide policy, and would only affect MMR coverage or overall exemptions, and not non-medical exemptions. In addition, these policies were unlikely to meaningfully affect our estimate since the calibration process of the synthetic control minimized these perturbations [39].** We plotted pre-policy trends in vaccination coverage and exemptions to assess the parallel trends assumption in the difference-in-differences analysis. Although the trends were similar between California and control counties before the intervention, there was greater variability in California over the years, especially for non-medical exemptions. Finally, our analyses included only two years of post-policy data, so we are unable to observe the persistence of increased coverage over time.

*Comment #2*

The analysis does not consider the other avenues for not up to date students to enter school in CA (conditional, not subject to immunization requirements).  And, unfortunately in this case, UTD rates is not an appropriate metric to evaluate the effectiveness of SB277 because efforts other than SB277 influenced UTD rate. Specifically, the analysis does not consider the statewide effort (that occurred one year prior to SB277) to reduce Conditional entrants, which resulted in a dramatic reduction of conditional entrants statewide and a corresponding increase in UTD rate (this can be seen in Fig 1 from 2014 to 2015). The Delamater et al. paper goes into great detail on this matter.

Response: We appreciate the reviewer’s attention to additional contributors to low vaccine coverage in this age group, particularly conditional entrants to kindergarten. The influence of policies such as SB277 on conditional enrollment has not been examined. Approximately 4% of students entering kindergarteners were conditional enrollees in 2015 (Buttenheim et al., 2018. <https://www.ncbi.nlm.nih.gov/pubmed/29778514>). Given the specific nature of this population, a more targeted examination would be needed to understand how policies such as SB277 influence these additional enrollment pathways. To address the reviewer’s comments we have included a note on conditional enrollment in our Limitations section.

-In Limitations.
**In 2015, California introduced a second initiative meant to educate administrators on the conditional admissions requirements. The 2015 conditional entrants initiative, while effective, was limited in scope compared to the statewide policy, and would only affect MMR coverage or overall exemptions, and not non-medical exemptions. In addition, these policies were unlikely to meaningfully affect our estimate since the calibration process of the synthetic control minimized these perturbations [39].**

*Comment #3*

The explanatory portion of the analysis seems somewhat underdeveloped, as the results in Table 1 are not really given much attention.

Response: We appreciate the reviewer’s interest in the correlates of vaccine coverage. We have added text to our Results section to further address the data presented in Table 1. We have also added further discussion.

-In Results:

In the county level analysis, we found that the trends for outcome variables were relatively similar between California counties and counties in control states in the pre-policy period (S5 Fig). Overall vaccination was generally similar in the pre-policy period, although California counties had slightly lower vaccination levels and greater variation than control counties. Pre-policy variation in non-medical exemptions for California was greater than for control states (S7 Table). In the county level analysis, we estimated that the California policy was associated with a 4.3% (95% CI 2.9-5.8, p<0.001) absolute increase in vaccine coverage for children entering kindergarten in California compared with those in control states (Table 1). The policy was also associated with a 3.9% (95% CI 5.4-2.4, p<0.001) absolute decrease in non-medical exemptions, and a 2.4% (95% CI 2.0-2.9, p<0.001) absolute increase in medical exemptions compared with counties in control states (Table 1). **Our model also estimated that income and education status of less than a high school degree were associated with increases in vaccination coverage and decreases in medical and non-medical exemptions (Table 1). Percent of uninsured children was significantly associated with a small increase in medical exemptions. Education status of some college as well as bachelor’s or beyond were significantly associated with small decreases in non-medical exemptions, while white race and percent of uninsured children were significantly associated with small increases in non-medical exemptions. (Table 1).**

-In Discussion

As multiple states continue to experience outbreaks of vaccine preventable diseases, interventions like the California policy that increase vaccine coverage levels above the required thresholds will continue to be vital. **A recent analysis of the California policy suggested that legislation may need to consider local differences, as areas of Northern California continued to demonstrate high rates of students without up-to-date vaccination status despite the state’s intervention [17]. Our county level analysis found a relationship between income and education and vaccine coverage, further demonstrating the complexity of this relationship. Additional research into other correlates of vaccine hesitancy in particular regions may be necessary to obtain ideal coverage in areas that remain vaccine hesitant.** Finally, settings that remain at low vaccine coverage despite removal of non-medical exemptions may require additional interventions that include local community involvement and educational programs [40-42].
