# Supplementary material for: The 2016 California policy to eliminate nonmedical vaccine exemptions and changes in vaccine coverage: An empirical policy analysis
Source: PLoS Med. 2019 Dec 23;16(12):e1002994. doi: 10.1371/journal.pmed.1002994 (PMC6927583; doi:10.1371/journal.pmed.1002994)
Supplement: Attachment — Submitted filename: Editor Response_2_Nov_14.docx [file pmed.1002994.s022.docx]

“The 2016 California Policy to Eliminate Non-Medical Vaccine Exemptions and Changes in Vaccine Coverage: An Empirical Policy Analysis” (your reference no. PMEDICINE-D-19-02566) – Point-by-point response

14 November 2019

Dear Editor,

We are pleased to submit a revised version of our Original Research article, entitled “The 2016 California Policy to Eliminate Non-Medical Vaccine Exemptions: An Empirical Policy Analysis” (your reference no. PMEDICINE-D-19-02566). We thank you for your helpful edits and comments, and for the invitation to submit a revised manuscript. We have addressed the issues brought forward and have included a point-by-point response below. Additionally, we have addressed the production issues sent to us by the publication team.

As policy makers in the United States and globally continue to tackle the growing challenge of vaccine hesitancy, we believe this article will inform policy making, in addition to making important contributions to the literature surrounding the efficacy of vaccine policies.

Sincerely,

Nathan C. Lo, MD PhD

University of California, San Francisco

On behalf of co-authors

**Response to Editors**

Comment #1

Thank you for pointing out your concerns over p value reporting and we do appreciate discussions in this area, however please do go ahead and provide p values as required by PLOS Medicine.

Response: We appreciate the Editor’s consideration about reporting of p-values. We have modified Table 1 in the main manuscript to include p-values as per PLOS Medicine’s requirement.

-In Results, Table 1

**Table 1: County level analysis of changes in vaccination coverage associated with the 2016 California policy using a difference-in-differences regression**

| **Parameter** | **Overall vaccination coverage (95% CI)^b^** | **p-value** | **Non-medical exemptions prevalence (95% CI)^b^** | **p-value** | **Medical exemptions prevalence (95% CI)^b^** | **p-value** |
| --- | --- | --- | --- | --- | --- | --- |
| **2016 California policy ^a^** | 4.3 (2.9 - 5.8) | <0.001 | -3.9 (5.4 - 2.4) | <0.001 | 2.4 (2.0 - 2.9) | <0.001 |
| **Median income per $10,000 (no)** | 1.0 (0.9 – 2.0) | <0.001 | -0.3(0.4 – 0.08) | 0.005 | -0.3 (0.4 - 0.1) | 0.002 |
| **Mean household size (no)** | -0.1 (-2.3 – 2.1) | 0.9 | 0.2 (-0.6 – 1.0) | 0.7 | 0.5 (-0.3 - 1.3) | 0.3 |
| **Population per 100,000 (no)** | -0.1 (-0.9 – 0.6) | 0.7 | 0.01 (-0.03 - 0.04) | 0.7 | 0.03 (-0.02 – 0.08) | 0.2 |
| **Poverty per 1,000 (%)** | -0.4 (-4.6 – 3.8) | 0.8 | -0.5 (-2.0 – 1.0) | 0.5 | 0.05 (-0.9 - 1.0) | 0.9 |
| **White (%)** | -0.03 (-0.06 – 0.01) | 0.2 | 0.03 (0.01 - 0.04) | 0.001 | 0.003 (-0.01 - 0.01) | 0.6 |
| **Education: Less than high school (%)** | 0.5 (0.2 - 0.7) | <0.001 | -0.3 (0.4 - 0.2) | <0.001 | -0.09 (0.1 - 0.04) | <0.001 |
| **Education: Some college or less (%)** | 0.2 (-0.03 - 0.4) | 0.1 | -0.2 (0.2 - 0.09) | <0.001 | -0.02 (-0.07 – 0.02) | 0.3 |
| **Education: Bachelor's degree or higher (%)** | 0.07 (-0.2 - 0.3) | 0.7 | -0.1 (0.2 - 0.09) | <0.001 | -0.01 (-0.06 – 0.05) | 0.8 |
| **Uninsured children (%)** | 0.08 (-0.01 - 0.2) | 0.1 | 0.09 (0.04 - 0.1) | <0.001 | 0.06 (0.04 - 0.08) | <0.001 |

Abbreviations: CI, Confidence Interval

^a^Difference-in-differences estimates represent relative change in county level vaccination and exemption prevalence for children entering kindergarten in California before and after the 2016 policy compared with children entering kindergarten before and after the policy in counties from control states

^b^Robust standard errors, clustered by county

Comment #2

In Introduction: Line 170: We suggest that you change the following sentence to read, “Following a series of high-profile exposures and outbreaks of vaccine-preventable diseases including a measles outbreak in Orange County, California [cite any supporting references], California passed Senate Bill 277 (SB277), which eliminated a previous policy that allowed non-medical exemptions to school immunization requirements [13-15].” or similar.

Specifically, please avoid attributing the measles outbreak to Disneyland, and please avoid implying that the implementation of SB277 was attributable to a single outbreak without providing a reference to support these statements. The citations provided in your response do not definitively establish that the measles outbreak originated in Disneyland (e.g. the report mentions that about one third of cases had unknown exposures), and also do not establish that the passing of the bill was the direct result of a single outbreak.

Response: We thank the Editors for their important note on correctly attributing the source of the 2015 measles outbreaks in California. We have removed the reference to Disneyland. We have also included additional citations for the statements made.

-In Introduction

Following a series of high-profile exposures and outbreaks of vaccine-preventable diseases including a measles outbreak in Orange County, California passed Senate Bill 277 (SB277), which eliminated a previous policy that allowed non-medical exemptions to school immunization requirements [13-17].

-In References

16.	Yang YT, Barraza L, Weidenaar K. Measles Outbreak as a Catalyst for Stricter Vaccine Exemption Legislation. JAMA. 2015;314(12):1229-30.

17.	Clemmons NS, Gastañaduy PA, Fiebelkorn AP, Redd SB, Wallace G. Measles - United States, January 4 - April 2, 2015. Morbidity and Mortality Weekly Report. 2015;64(14):373 - 6.

Comment #3

Title: Please revise to: “Estimated changes in vaccine coverage associated with the 2016 California policy to eliminate non-medical exemptions: An empirical policy analysis” or similar, to make it clear what you are evaluating in your study (i.e. your study outcome).

Response: We thank the editors for the attention to our title. We have modified the title as suggested above.

-In Title

**“The 2016 California Policy to Eliminate Non-Medical Vaccine Exemptions and Changes in Vaccine Coverage: An Empirical Policy Analysis”**

Comment #4

Abstract: Background: Please revise the final sentence to “The objective of this study was to estimate the association between California’s 2016 policy and vaccine coverage.” or similar to avoid any suggestion of causality.

Response: We thank the authors for their attention the casual implications of the verbiage used in our abstract. We have modified the abstract as suggested above to remove any causal phrases.

-In Abstract

State policies that eliminate non-medical (“personal belief”) exemptions to childhood vaccination requirements are controversial, and their effectiveness to improve vaccination coverage remains unclear given limited rigorous policy analysis. In 2016, a California policy (Senate Bill 277) eliminated non-medical exemptions from school entry requirements. **The objective of this study was to estimate the association between California’s 2016 policy and vaccine coverage.**

Comment #5

Abstract: Conclusions: Please revise the first sentence to: “In this study, implementation of the California policy was associated with an estimated increase in vaccination coverage and a reduction in non-medical exemptions at state and county levels.”

Response: We thank the authors for their attention the language used in our Abstract. We have modified the abstract as suggested above.

-In Abstract

**Conclusions: In this study, implementation of the California policy was associated with an estimated increase in vaccination coverage and a reduction in non-medical exemptions at state and county levels.** The observed increase in medical exemptions was offset by the larger reduction in non-medical exemptions.

Comment #6

Summary Points: Please remove the section called “Summary points” from the manuscript.

Response: We have removed the first set of summary points from the manuscript.

Comment #7

Author Summary: “Why was this study done?”: Please revise the second bullet point to: “...restrict the ability of parents to obtain non-medical exemptions from vaccination requirements…”

Response: We thank the Editors for their attention to the language used in our summary points. We have modified the summary points to use the suggested language.

-In Author Summary

Limited empirical research has evaluated the impact of state vaccination policies that **restrict the ability of parents to obtain non-medical exemptions from vaccination requirements.**

The 2016 California vaccine policy (Senate Bill 277) that eliminated non-medical childhood vaccination exemptions provides an opportunity to empirically estimate the impact of these policies on vaccine coverage.

Comment #8

Author Summary: “Why was this study done?”: Please revise the third bullet point to: “The 2016 California vaccine policy (Senate Bill 277 (SB277) that eliminated non-medical childhood vaccination exemptions provides an opportunity to empirically estimate the impact of these policies on vaccine coverage.”

Response: We have revised the author summary to include the language suggested above.

-In Authors Summary.

- Limited empirical research has evaluated the impact of state vaccination policies that restrict the ability of parents to obtain non-medical exemptions from vaccination requirements.
- **The 2016 California vaccine policy (Senate Bill 277) that eliminated non-medical childhood vaccination exemptions provides an opportunity to empirically estimate the impact of these policies on vaccine coverage.**

Comment #9

Introduction: Lines 182-184: Please revise the final sentence of the introduction to: “To address ongoing debate surrounding these governmental policies, we provide a quasi-experimental, controlled analysis to evaluate the association of California’s policy and vaccination and exemption outcomes.” to avoid the suggestion of causality.

Response: We have modified the language in the Introduction section as suggested above.

-In Introduction

The aim of our study was to conduct a rigorous, controlled analysis to estimate the effectiveness of California’s policy restricting non-medical exemptions to increase vaccination coverage. **To address ongoing debate surrounding these governmental policies, we provide a quasi-experimental, controlled analysis to evaluate the association of California’s policy and vaccination and exemption outcomes.**

Comment #10

Methods: Line 188-189: Please revise the first sentence of the methods to: “We performed two empirical analyses to estimate the association of the 2016 California policy with changes in vaccination coverage and exemptions…”

Response: We have modified the language in the Methods section as suggested above.

-In Methods

**We performed two empirical analyses to estimate the association of the 2016 California policy with changes in vaccination coverage and exemptions:** 1) a synthetic control analysis, where we used state level data to create a hypothetical counterfactual “synthetic control California” to compare to the real California; and 2) a difference-in-differences analysis, where we used county level data.

Comment #11

Methods: Line 206-207: Please revise the sentence to: “We included medical and non-medical exemptions as outcomes to evaluate if any compensatory increases in medical exemptions were observed to be associated with the policy.”

Response: We have modified the language in the Methods section as suggested above.

The secondary outcomes were the proportion of children entering kindergarten with medical and non-medical exemptions at the start of the school year**. We included medical and non-medical exemptions as outcomes to evaluate if any compensatory increases in medical exemptions were observed to be associated with the policy.** We used a range of publicly available state covariate data (S1 Table).

Comment #12

Methods: Line 225: Please change “DtaP” to DTaP”.

Response: We have changed “DtaP” to DTaP”.

-In Methods.

The immunizations required to enroll in kindergarten vary by state but generally include MMR, polio, chickenpox (varicella), and diphtheria, tetanus, and pertussis **(DTaP)** [28].

Comment #13

Discussion: Lines 475-478: Please revise this sentence to: “In this study, in both the county and state level analyses, non-medical exemptions decreased and medical exemptions increased, a trend that supports our conclusion that the observed increase in vaccination coverage in California is associated with the implementation of the 2016 policy rather than confounding factors.” to avoid any implications of causality.

Response: We have modified the text to remove causal language as suggested.

-In Discussion

**In this study, in both the county and state level analyses, non-medical exemptions decreased and medical exemptions increased, a trend that supports our conclusion that the observed increase in vaccination coverage in California is associated with the implementation of the 2016 policy rather than confounding factors.** Additionally, although the rise in medical exemptions could indicate that some children who may have received non-medical exemptions in the past are now receiving medical exemptions, the net effect following the California policy was still an increase in vaccination coverage.

Comment #14

Fig 1: Please revise the Figure title to: “State level analysis of the 2016 California policy using synthetic control methodology on childhood vaccination and exemption outcomes”

Response: We have revised the Fig. 1 title as suggested.

**Fig 1: State level analysis of the 2016 California policy using synthetic control methodology on childhood vaccination and exemption outcomes.** We constructed a synthetic control state for California for three study outcomes of measles, mumps, and rubella (MMR) coverage (top), non-medical exemptions (bottom left), and medical exemptions (bottom right).

Comment #15

Fig 3: Please revise the Figure title to: “Placebo testing for the state level analysis of 2016 California policy using synthetic control methodology”

Response: We have revised the Fig. 3 title as suggested.

**Fig 3: Placebo testing for the state level analysis of the 2016 California policy using synthetic control methodology.** Placebo testing is used in synthetic control analyses to determine whether an identified relationship is statistically meaningful.

Comment #16
Supplemental Information: S3 and S4 Tables: Thank you for defining the abbreviation for “BS”. In reference to the abbreviation “BS” for “bachelor’s degree”- please consider that use of BS may imply individuals holding a bachelor’s of science degree. Would it be a more accurate reflection of your study data to simply refer to a bachelor’s degree generally?

Response: We thank the editor for their attention to this issue. We have modified the text in S3 and S4 Tables to include Bachelor’s Degree, to avoid any confusion about the meaning of “BS”.

-In S3 Table

**S3 Table: Characteristic covariate weights used to create the synthetic California for each outcome in base case analysis**

| **MMR Coverage** | | **Non-medical Exemptions** | | **Medical Exemptions** | |
| --- | --- | --- | --- | --- | --- |
| **Covariate** | **Weight**  **(%)** | **Covariate** | **Weight**  **(%)** | **Covariate** | **Weight**  **(%)** |
| Average MMR Lag | 96 | Average MMR Lag | 90 | Average MMR Lag | 98 |
| Median Age | 0.01 | Median Age | 8.7 | Private Insurance | 2 |
| No Well Child Visit | 2.6 | Population | 0.4 | Per Capital Health | 0 |
| Uninsured | 0.7 | Education, Bachelor’s degree | 0.4 |  |  |
| Private Insurance | 1.2 | No Coverage | 0.07 |  |  |
| Population | 0.9 |  |  |  |  |

Abbreviations: MMR, Measles Mumps and Rubella Vaccine.

-In S4 Table

**S4 Table: Characteristic covariate sensitivity analysis for state level synthetic control analysis**

| **Covariate** | **MMR Coverage** | **Covariate** | **Non-medical Exemptions** | **Covariate** | **Medical Exemptions** |
| --- | --- | --- | --- | --- | --- |
| Average pre-policy lag | 3.23 | Average pre-policy lag | -2.47 | Average pre-policy lag | 0.39 |
| Median Age | 3.34 | Median Age | -2.64 | Private Insurance | 0.39 |
| No Well Child Visit | 3.47 | Population | -2.37 | Per Capita health expenditure | 0.39 |
| Uninsured | 3.62 | Education, Bachelor’s degree | -2.78 | Median Age | 0.40 |
| Private Insurance | 3.34 | No Coverage | -2.36 | Median Income | 0.40 |
| Population | 3.18 | Live Rural | -2.37 | Children with no Insurance | 0.35 |
| Per Capita health expenditure | 3.49 | Per Capita health expenditure | -2.42 | No Previous Well Child Visit | 0.38 |
| Children with no Insurance | 3.13 | Children with no Insurance | -2.49 | No Coverage | 0.36 |
| Median Income | 3.40 | Below Poverty | -2.22 | Married | 0.38 |
| All Covariates | 3.23 | All Covariates | -2.36 | All Covariates | 0.36 |
| **Reported [Range]** | **3.34**  **[3.13 - 3.62]** |  | **-2.36**  **[2.78-2.22]** |  | **0.39**  **[0.35 - 0.40]** |

Abbreviations: MMR, Measles Mumps and Rubella Vaccine.

**Production issues:**

Metadata

-       PLOS requires corresponding authors to have an ORCID iD. Please ensure that Dr. Lo, if he is to be the corresponding author on the manuscript, has an ORCID iD and that it is validated in Editorial Manager system. To do this, he can go to ‘Update my Information’ (in the upper left-hand corner of the Editorial Manager main menu), and click on the Fetch/Validate link next to the ORCID field. This will take you to the ORCID site and allow you to create a new iD or authenticate a pre-existing iD in Editorial Manager. For more information please visit: <https://www.plos.org/orcid>. We cannot proceed with the manuscript unless the corresponding author has a linked ORCID.

Response:

**The corresponding author (Dr. Nathan Lo) has an ORCID: 0000-0002-5407-2005. This is linked to his PLOS profile.**
